# Unveiling Strong Thin Film Confinement Effects on Semirigid Conjugated Polymers

**DOI:** 10.1021/acs.macromol.4c01500

**Published:** 2024-09-23

**Authors:** Haoyu Zhao, Zhaofan Li, Yunfei Wang, Qi-An Hong, Wenjie Xia, Yu-Cheng Chiu, Xiaodan Gu

**Affiliations:** † School of Polymer Science and Engineering, The University of Southern Mississippi, 118 College Drive, Hattiesburg, Mississippi 39406, United States of America; ‡ Department of Aerospace Engineering, Iowa State University, Ames, Iowa 50011, United States of America; § Department of Chemical Engineering, National Taiwan University of Science and Technology, Taipei City 10607, Taiwan

## Abstract

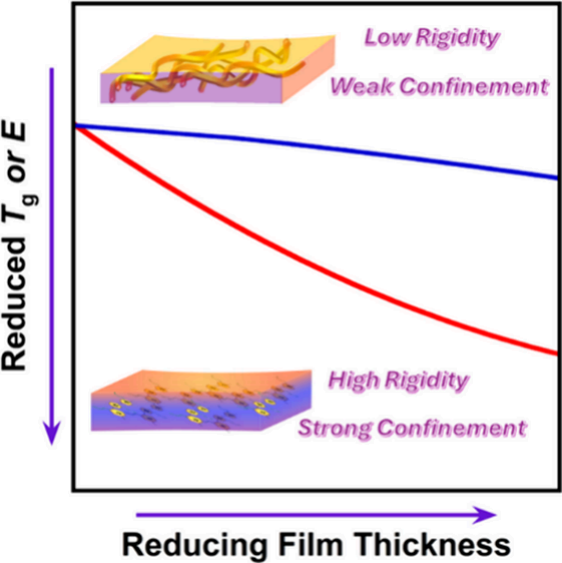

Nanoconfinement has been recognized to induce significant changes in the physical properties of polymeric films when their thickness is less than 100 nm. Despite extensive research on the effect of nanoconfinement on nonconjugated polymers, studies focusing on the confinement effects on dynamics and associated electronic and mechanical properties for semiconductive and semirigid conjugated polymers remain limited. In this study, we conducted a comprehensive investigation into the nanoconfinement effects on both p- and n-type conjugated polymers having varying chain rigidity under different degrees of confinement. Using the flash differential scanning calorimetry technique, it was found that the increased molecular mobility with decreasing film thickness, as indicated by the depression of glass transition temperature (*T*_g_) from its bulk values, was directly proportional to chain rigidity. This relationship between chain rigidity and enhanced segmental mobility was further corroborated through molecular dynamics simulations. Thinner films exhibited a higher degree of crystallinity for all conjugated polymers, and a significant reduction of more than 50% in elastic modulus was observed for films with approximately 20 nm thickness compared to those of 105 nm thickness, particularly for highly rigid conjugated polymers. Interestingly, we found that the charge mobility remained independent of film thickness, with all samples demonstrating good charge mobility regardless of the different film thicknesses for devices measured here. Nanoconfined conjugated polymer thin films exhibited a combination of mechanical compliance and good charge carrier mobility properties, making them promising candidates for the next generation of flexible and portable organic electronics. From an engineering standpoint, confinement could be an effective strategy to tailor the dynamics and mechanical properties without significant loss of electronic property.

## Introduction

The discovery of inorganic silicon-based semiconductor materials and transistors has greatly transformed human being’s daily life. However, due to the restrictions in deformability and energy-intensive synthesis of single crystal silicon and costly processing for inorganic semiconductors, organic electronics emerged as a new class of materials that meets the increasing demands of emerging applications that require portable, flexible, and even biodegradable capability.^1,2^ Conjugated polymers (CPs), organic macromolecules consisting of π-functional backbone and highly flexible side chains, not only being soft and deformable but also own unique optoelectronic properties.^3^ After the initial success of the thiophene-based first-generation CPs, the donor–acceptor (D-A) polymers have been widely studied in the field, as they demonstrate significant improvements in both electronic and optical properties. A sophisticated molecular design has been added to these macromolecules, with the goal of promoting enhancements in intra/interchain charge transport through fine-tuning of backbone rigidity, aggregation/alignment, and packing structure.^4−6^ Since the active layers for most organic electronic devices are below 100 nm, it is important to understand the effect of the confinement on thin film morphology and dynamics of those novel polymers.

Glass transition temperatures (*T*_g_), an important physical parameter for amorphous and semicrystalline materials that describe the segmental mobility, determine the applicable operation temperature for a given polymer, as well as their physical/mechanical properties, and suitable processing conditions. The studies of the glass transition phenomenon have been conducted for more than half a century; however, the resolution of the glass problem in solid state theory still remains unsolved despite the many efforts made during the past three decades.^7^ Pioneering work by Keddie et al.^8^ in the early 90s revealed that the *T*_g_ of sub-100 nm thin film would deviate from its bulk *T*_g_ value, where changes of *T*_g_ upon confinement are influenced by interactions between polymer and substrate. Intensive work has been done for nonconjugated systems, with a focus on the nanoconfinement effects on polymers characterized by multiple techniques.^9−13^ In general, for supported thin film, most polymers showed reduced *T*_g_ if there is no strong interaction between the film and the substrate. On the other hand, for free-standing thin film,^14,15^ molecular weights played an important role in depression rates that high molecular weights free-standing film exhibited linear reduction trends but low molecular weight counterpart is more easily subjected to the film–substrate interactions.

Contrary to conventional polymers, the characterization of *T*_g_ of CPs is much more challenging due to the fact of complex backbone structures and overall heterogeneous dynamics.^16^ For nonconjugated polymers, the conformational changes before/after glass transition are substantial where the heat capacity changes between glassy and liquid lines are in the range of 0.1–0.8 J g^–1^ K^–1^, so that differential scanning calorimetry (DSC) can easily capture such heat variations.^17^ Nonetheless, conjugated polymers’ backbones are much more rigid, so the conformational changes are restricted by highly rigid chains near the glass transition. In addition, the partial crystallinity introduced by CP semicrystalline nature hinders thermal signals, resulting in less than 0.05 J g^–1^ K^–1^ for heat capacity changes,^16^ which is beyond the resolution of conventional DSC. Another commonly adopted technique for conventional polymers’ *T*_g_ measurements is dynamic mechanical analysis (DMA), which monitors the storage and loss modulus changes upon temperature history to deduce the dynamical *T*_g_. However, the sample mass requirement of gram level becomes the major obstacle for applying the DMA technique to conjugated polymers,^18^ since those materials are normally synthesized in small batches using the cross-coupling reaction. Additionally, the DMA measurement is typically for bulk samples. Thus, the difficulties in quantitative detections of thin film *T*_g_ are the main reasons for a lack of reported *T*_g_ values for nanoconfined CP films in literature. Therefore, there remains a knowledge gap in understanding the effect of nanoscopic confinement on the chain dynamics of CPs.

Additionally, it is important to understand the effect of nanoconfinement on *T*_g_ for organic semiconductor applications. The flexible and deformable organic electronics require relatively low elastic modulus, especially for wearable devices where elastic modulus close to human skins of a few megaPascals (MPa) is mandatory. To achieve that, the processed thin film is expected to be maintained in the vicinity of or above its bulk *T*_g_ since elastic modulus could decrease by several orders of magnitude from glassy to rubbery states.^19^ Therefore, the thermomechanical properties of conjugated polymers should be investigated to explore the optimum film thickness conditions and the processing approach to suppress *T*_g_ and reduce the elastic modulus.

Due to the limitations of thin film geometries, the mechanical properties of thin films have been studied using bulking metrology and the film floated on the water surface method. Stafford et al. first developed a buckling-based metrology targeting thin film polymers with film thickness ranging from 10 to 100 nm.^20−22^ In this method, an elastic predeformed (<2% strain) substrate (poly(dimethylsiloxane)) is required to support the testing thin film. The loaded stress will cause energetic competition between bent film and deformed substrate resulting in periodic wrinkles. The periodicity of the bulked film is interpreted into elastic modulus by surface metrology instruments. This method was successfully applied on CP thin films;^23,24^ however, the shortcomings of this method included the interactions between testing film and substrates and not quantified strain rate. Due to the presence of a supported substrate, this method is categorized as an indirect method, where the interactions between polymer and substrate could contribute to the quantifications of testing film mechanical properties. In addition, the unspecified strain rates lead to challenges for viscoelastic thin film mechanical properties due to the molecular relaxations over time. On the other hand, the pseudo free-standing tensile test used in this study is a direct method for stress–strain responses for free-standing semiconducting polymeric thin films, which was inspired by the Crosby group.^25^ This method is not influenced by polymer/substrate interactions, and it enables several new measurements, which are not achievable from buckling metrology, for example, stress–strain dependence and hysteresis tests. Nonetheless, this method requires the thin film to float on the water surface so that the influences of absorbed water for certain polymers were investigated and discussed in our group's previous work.^26^

Moreover, the optoelectronic properties are apparently affected by nanoconfinement as well due to changes in morphology and chain mobility. For organic thin film, once the film thickness is reduced below 100 nm, the surface effects, the interior bulk film, and the bottom film/substrate interface effects all play significant roles in the determination of morphological changes (e.g., crystallinity, orientation, alignment) and dynamical variations (e.g., enhanced mobility due to free surface effects, slower mobility due to strong interaction between interface interactions, steady mobility due to the competition between surface and interface effects). For instance, the organic photovoltaic devices (OPV) need to maintain the phase separation size to efficiently harvest excitons and separate charges.^27^ However, the long-term operations above *T*_g_ of the active layer will result in severe phase separation toward performance loss. For organic field-effect transistors (OFETs), short-period thermal annealing above film *T*_g_ can facilitate ordered morphology but the increased mobility could induce the dynamic disorder near the interface leading to reduced charge carrier mobilities.^28,29^ As such, one can see that the confinement effect is ubiquitous and an important consideration for organic electronics.

For the scope of this article, we selected a few representative CPs to investigate the nanoconfinement effects. We first quantified the dependence of *T*_g_ on film thickness for CPs using the thin film fast scanning differential calorimetry (Flash DSC). This specialized thin film technique was employed to measure *T*_g_ for a film thickness ranging from 40 to over 100 nm. We observed that *T*_g_ of CP thin film was reduced as film thickness decreased, where the difference between confined and bulk *T*_g_ was associated with CP backbone rigidity. The relationship between the *T*_g_ depression and chain rigidity was further verified by molecular dynamics (MD) simulations. Additionally, a lower elastic modulus was observed at thinner film thickness by our unique pseudo free-standing tensile testing for confined films below 100 nm. Finally, the charge carrier mobility under confinement was carefully examined and no significant mobility loss was found, indicating that the thin film transistor could maintain its charge carrier mobility compared to unconfined film. Within the understanding of nanoconfinement on molecular mobility, thermomechanical properties, and optoelectronic properties, our study will provide a pathway to finely tune the molecular structures and film thickness to meet the requirements for the next generation of flexible organic electronics.

## Experimental Section

### Materials

Regioregular poly(3-hexylthiophene-2,5-diyl) (P3HT) was purchased from Rieke Materials with regioregularity higher than 90% (Mn: 26 538 g/mol, PDI: 2.6). Poly{2,2′-[(2,5-bis(2-hexyldecyl)-3,6-dioxo-2,3,5,6- tetrahydropyrrolo[3,4-c]pyrrole-1,4-diyl)dithiophene]- 5,5′-diyl-*alt*-thiophen-2,5-diyl}(PDPPT) (*M*_n_: 34 736 g/mol, PDI: 1.8), poly[(2,6-(4,8-bis(5-(2-ethylhexyl-3-fluoro)thiophen-2-yl)-benzo[1,2-b:4,5-b′]dithiophene))-*alt*-(5,5-(1′,3′-di-2-thienyl-5′,7′-bis(2-ethylhexyl)benzo[1′,2′-c:4′,5′-c′]dithiophene-4,8-dione)] (PM6) (*M*_n_: 60 551 g/mol, PDI: 2.1) and poly{[N,*N*′-bis(2-hexylldecyl)naphthalene-1,4,5,8-bis(dicarboximide)-2,6-diyl]-*alt*-2,5-thiophene}(NDI(2HD)T) (*M*_n_: 148 132 g/mol, PDI: 1.8) were purchased from Ossila Inc. All CPs were dissolved in chlorobenzene (CB) at a temperature of 80 °C with a magnetic stirrer for 8 h to form a homogeneous solution. The solution was then filtered and spin-coated with spin speed ranges from 1000 to 4000 rpm to prepare the thin films. The thin film was then transferred using the water transfer technique^30^ onto the surface of the Flash DSC chip membrane for the following characterizations.

### Characterization Techniques

#### Differential Scanning Calorimetry

A Mettler–Toledo Flash differential scanning calorimeter (Flash DSC 2+) was used for the *T*_g_ measurement of conjugated polymers. The ultrafast standard chip with heating and cooling rates up to 4000 K/s was employed in Flash DSC. The testing films were held for 5 s at the melting state to erase the previous thermal history and then cooled at 0.1 K/s, where *T*_g_ was obtained by the subsequent heating scans. All measurements were done under nitrogen gas with a flow rate of 60 mL/min at ambient pressure. The film thickness of the testing specimen was recorded by a Bruker DektakXT stylus profilometer with a stylus tip radius of 12.5 μm using standard hills and valleys scan for the maximum range of 524 μm over a 2 mm-long area.

#### Molecular Dynamics Simulation

Within our established coarse-grained (CG) modeling framework,^31^ we present the atomistically informed CG model for P3HT as illustrated in Figure 2a. In this representation, each P3HT monomer is characterized by three distinct types of CG beads (i.e., P1, P2, and P3), corresponding to the thiophene ring, the first three consecutive hexyl side chain methyl groups, and the last three hexyl side chain methyl groups, respectively. The force center is positioned at the center of mass of the atoms constituting the underlying CG bead. The CG force field for P3HT employed in this study was systematically derived in our earlier work through the energy renormalization (ER) approach. This temperature-transferable CG model effectively captures the glass-forming dynamics across a broad temperature range, demonstrating its utility in coarse-grained simulations. More detailed information on the CG model development and application can be referenced in our previous studies.^31,32^ Within our methodology, bending constraints are typically applied to three successive beads along the polymer chain. To enhance the bending rigidity, we introduce a rescaling factor, *K*_rigidity_, for angular stiffness, allowing us to control the rigidity of the polymer chain’s backbone. Specifically, when *K*_rigidity_ is set to 1, the CG model corresponds to P3HT. In contrast, when *K*_rigidity_ is increased to 2 or 4, it serves to emulate more rigid CPs. This strategic manipulation enables us to systematically explore the impact of rigidity on the dynamics and mechanical behaviors of the polymer system.

Large-scale Atomic/Molecular Massively Parallel Simulator (LAMMPS) simulation package was applied to perform all CG-MD simulations,^33^ and the visualization of simulation snapshots was performed through the Visual Molecular Dynamics (VMD).^34^ In simulating the bulk system, we randomly packed models consisting of backbone chains, each composed of 20 CG beads, into the simulation cell. Periodic boundary conditions (PBCs) were applied in all directions, and a time step of Δ*t* = 4 ps was employed to integrate the equations of motion, optimizing computational efficiency. To achieve system equilibration, energy minimization was initially conducted using the iterative conjugate gradient algorithm.^35^ Subsequently, the system underwent equilibration in the melt state at a high temperature (1000 K) under the isothermal–isobaric (NPT) ensemble for 2.5 ns, with pressure ramping from an initial 1000 atm to a final 100 atm. Following this, the system was gradually cooled to various target temperatures at 1 atm pressure under the NPT ensemble for 4 ns before data sampling.

For additional thin film simulations, PBCs were applied in both the *x*- and *y*-directions, defining dimensions of 8 nm × 8 nm, while non-PBCs were applied in the *z*-dimension (thickness direction). To simulate a supported film, a completely smooth and implicitly attractive wall was introduced as the substrate at the lower *z* face in the *x*–*y* plane of the simulation box. The implemented attractive wall features a truncated Lennard–Jones (LJ) 12–6 potential form, denoted as , where *z* represents the distance from the polymer to the substrate, ε_sp_ is the energy strength of attraction to the substrate, and σ_sub_ is the distance at which the attraction is zero. It is important to note that the choice of ε_sp_ in this study is very small and nearly close to zero, mimicking the very weak interaction between the polymer system and the substrate, consistent with the experimental setup. In the special case where ε_sp_ = 0 kcal/mol, the system becomes a free-standing thin film with no substrate interaction. The center of the film was aligned with the *x*–*y* plane at *z* = 0. Subsequently, a similar equilibrium process was adopted for MD simulations under the canonical ensemble (NVT). Three models were generated with independent initial configurations for each system, ensuring sufficient sampling for determining averaged properties along with their standard deviations.

In our analysis of the dynamics, we commenced with the investigation of the structural relaxation time τ_α_, a pivotal dynamic parameter in glass formation. The determination of τ_α_ involved the intermediate scattering function *F*_*s*_(*q*, *t*),
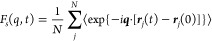
1where *N* is the number of beads in the system, *q* = |***q***| is the wavenumber derived from the first peak position of structure factor *S*(*q*), ***r***_*j*_(*t*) is the position of the *j*th bead at time *t*, and angular brackets denote the ensemble average. The calculation of *F*_*s*_(*q*, *t*) allows us to assess τ_α_, conventionally defined as the time at which *F*_*s*_(*q*, *t*) decays to 0.2.^36^

The Debye–Waller factor, ⟨*u*^2^⟩, serves as a measure of molecular “free volume” and “stiffness” on the order of a picosecond time scale.^37^ Experimentally, ⟨*u*^2^⟩ is obtainable through X-ray and neutron scattering techniques.^38,39^ In MD simulations, we define ⟨*u*^2^⟩ as the plateau value of the mean-square displacement (MSD) ⟨*r*^2^(*t*)⟩ for all CG beads at approximately *t* = 4 ps, a time scale associated with the onset of caging, consistent with previous simulations.^31,40^ The calculation of ⟨*r*^2^(*t*)⟩ involves:
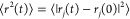
2where *r*_*j*_(*t*) is the position of the *j*th bead at time *t*, and ⟨*r*^2^(*t*)⟩ is obtained from the average of all beads in the system.

To assess the mechanical properties of the thin film model, nonequilibrium tensile deformation was conducted with a constant strain rate of 0.5 ns^–1^, aligning with the range commonly utilized in previous studies.^41−43^ The tensile modulus was determined by fitting a linear slope to the stress–strain curves in the range of strain of ≤4%. The stress components of the system were calculated using the atomic virial stress tensor:
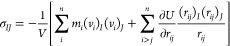
3where *V* represents the system volume, *n* is the total number of beads, *r*_*ij*_ stands for the distance between beads *i* and *j*, *U* is the total energy of the system, and *m*_*i*_ and *v*_*i*_ are the mass and velocity of the bead *i*, respectively. Specifically, the equation for the *I*, *J* components (where *I* and *J* = *x*, *y*, *z*) refers to the 6-element symmetric stress tensor. The tensile stress component is determined as σ_*xx*_.

#### X-ray Scattering

GIWAXS analysis was conducted using the Xenocs Xeuss 2.0 beamline equipped with a Pilatus 1 M detector, utilizing an X-ray wavelength of 1.54 Å. The setup included a sample-to-detector distance of 150 mm. The critical angle of our systems is close to the critical angle of PS (∼0.15) and the incident angle is 0.2 for our measurements. Thus, the X-ray beam will penetrate the sample but not the silicone substrate due to the critical angle of silicon of 0.22°. Data acquisition and processing were facilitated through Igor Pro version 8 software, employing the Nika package and WAXSTools for data manipulation. The measurements for rDOC were developed by Baker et al. and Toney et al.^44,45^ The rDOC for each sample was derived from the normalized intensity of the (100) peak, adjusting for the exposure time, sample thickness, and path length of the beam. Subsequently, a geometric correction to the orientation distribution function, represented as sin(χ)*I*(χ), was applied to evaluate the relative orientation of the crystallites. The degree of crystallinity was quantified by integrating the area under each pole figure curve. The data processing method and its underlying mechanism were reported in the literature.

#### Mechanical Properties Testing

Mechanical characterization was conducted using a pseudo free-standing tensile test, where thin films were tested on the surface of water.^46^ The testing temperature range is from 10 to 60 °C. Specifically, polymer films were patterned into a dog-bone shape by using a laser patterning method and then transferred onto water. These films were subsequently stretched unidirectionally at a strain rate of 5 × 10^–3^ s^–1^ until fracture occurred. Five independent tests were carried out for each conjugated polymer at each specified thickness to ensure statistically reliable mechanical properties. The elastic modulus was determined from the initial slope of the stress–strain curve, considering only the first 0.5% of strain, which corresponds to the elastic region. The crack onset strain (COS) was identified as the strain at which initial cracking was observed.

#### Charge Carrier Mobility

CPs were separately dissolved overnight in CB at 70 °C. In the N_2_-filled glovebox, the solution was spin-coated onto the OTS-treated 300 nm SiO_2_/Si substrate (capacitance per unit area Ci = 10 nF cm^–2^) at different spin-coated speeds and concentrations to control the semiconductor film thickness. Afterward, under the high vacuum conditions with pressure below 10^–6^ Torr, the gold electrodes of 50 nm thickness were thermally deposited at a rate of 0.5 Å/s and a shadow mask with a width-to-length ratio of the drain and source electrodes of 1000 and 50 μm, respectively. Finally, the electrical performance was measured using a Keithley 4200-SCS for the transistor structure with a bottom-gate top-contact at room temperature in a N_2_-filled glovebox, and the carrier mobility (μ_sat_) and the threshold voltage (*V*_th_) were calculated based on the saturation region. In addition, the thickness of CP films for device mobility calculation was measured by AFM.

## Results

In this work, we studied a comprehensive range of the CP’s physical behavior in a confined state with different thicknesses. This includes the chain mobilities, morphology, and mechanical and electrical properties of those CPs. To systematically investigate the influences of confinement effects, we have selected a few representative P-type and N-type CPs with different backbone rigidities.^47,48^ The classic P3HT has flexible backbones with a persistence length (*L*_p_) of 3 nm,^49,50^ and the high-performance N-type poly{[*N*,*N*′-bis(2-hexylldecyl)naphthalene-1,4,5,8-bis(dicarboximide)-2,6-diyl]-*alt*-2,5-thiophene} (PNDI) owns the highest *L*_p_ of 22 nm, representing the most rigid backbone in this study. The high-performance OFET material of poly{2,2′-[(2,5-bis(2-hexyldecyl)-3,6-dioxo-2,3,5,6- tetrahydropyrrolo[3,4-c]pyrrole-1,4-diyl)dithiophene]-5,5′-diyl-*alt*-thiophen-2,5-diyl} (PDPPT) and OPV donor, poly[(2,6-(4,8-bis(5-(2-ethylhexyl-3-fluoro)thiophen-2-yl)-benzo[1,2-b:4,5-b′]dithiophene))-*alt*-(5,5-(1′,3′-di-2-thienyl-5′,7′-bis(2-ethylhexyl)benzo[1′,2′-c:4′,5′-c′]dithiophene-4,8-dione)) (PM6), has *L*_p_ values of 15 and 17 nm, respectively.^47,48,51^ The chain mobility is characterized by the backbone *T*_g_ of CPs since the *T*_g_ determines the lowest temperature for the backbone segmental motions. Considering that the film thickness is in the sub-100 nm range, flash DSC was conducted to export thermodynamic *T*_g_ of selected CPs to overcome the limitations of *T*_g_ measurements using conventional DSC.^16^

### Confinement Effect on *T*_g_

We first demonstrated the effectiveness of Flash DSC in determining the *T*_g_ for nanoconfined CP thin film. *T*_g_ is a kinetic phenomenon that can shift to a higher or lower temperature depending on the heating/cooling rates. Conventionally, the *T*_g_ value is normally obtained from a subsequent heating scan after specific cooling rates. Such a temperature profile could enhance measurement signal and resolution.^40^ Here, typical measurements used a fixed heating rate and varying cooling rates to investigate the relaxation dynamics. As the cooling rate decreases, an endothermic peak in the transition area becomes more appreciable, and the onset of such peak also shifts to high temperature, known as enthalpy overshoot.^7,10^ The appearance of enthalpy overshoot is attributed to reduced molecular dynamics at lower cooling rates, and a higher temperature is required to reach the equilibrium liquid line for any given heating scans.^7^ Therefore, by applying a greater mismatch between heating (denoted *m*) and cooling rates (denoted *q*), the occurrence of enthalpy overshoot provides a clear thermal signature to locate the glass transition regions of CP’s rigid backbone.

**Figure 1:**
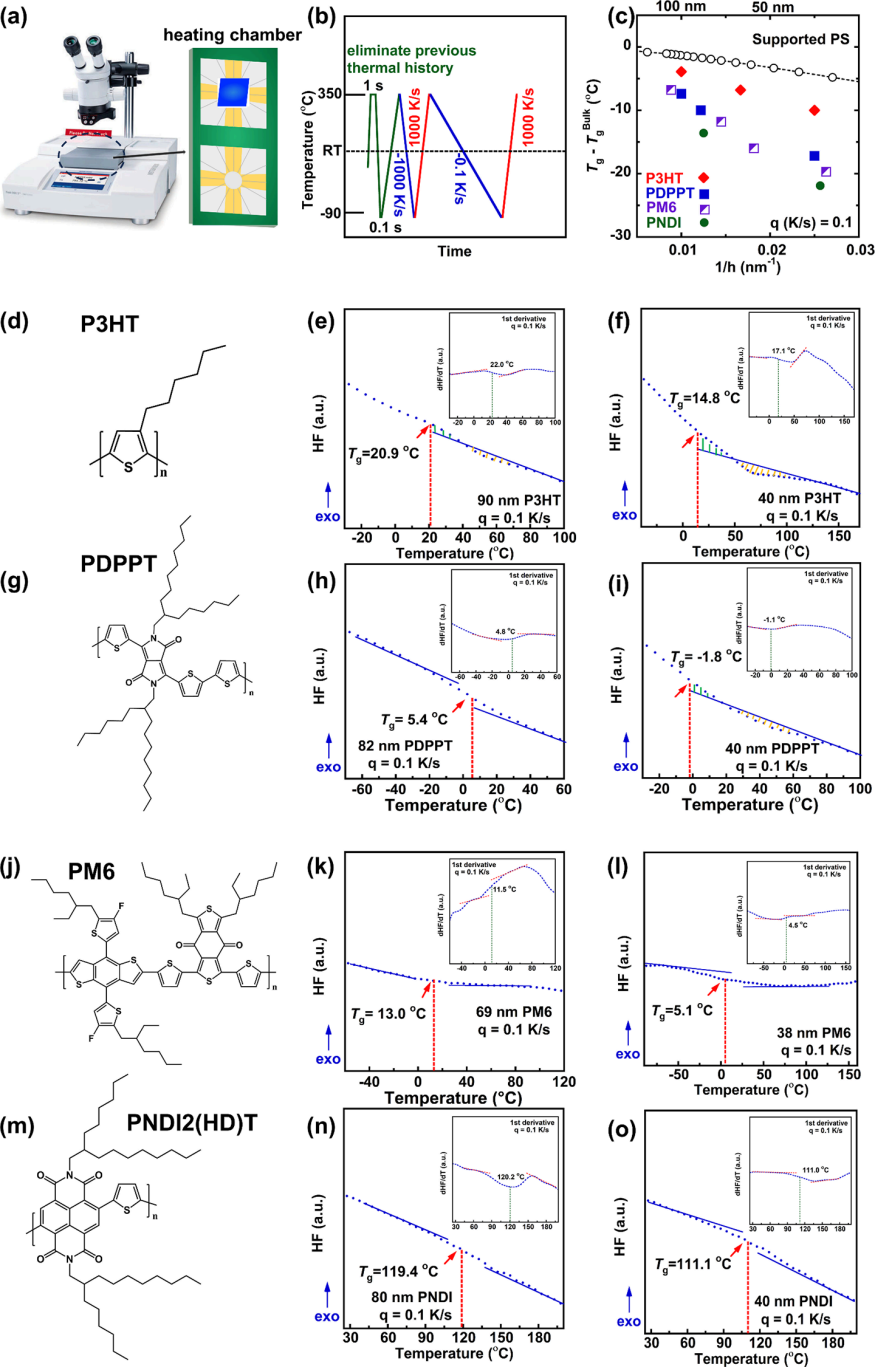
Chain mobility for various conjugated polymers. (a) Flash DSC instrument and heating chamber for thin film. (b) Flash DSC temperature protocols. (c) Confinement strength as a function of inverse of film thickness. (d) P3HT’s molecular structure. (e) *T*_g_ of 90 nm P3HT. (f) *T*_g_ of 40 nm P3HT. (g) PDPP’s molecular structure. (h) *T*_g_ of 82 nm PDPPT. (i) *T*_g_ of 40 nm PDPPT. (j) PM6’s molecular structure. (k) *T*_g_ of 69 nm PM6. (l) *T*_g_ of 38 nm PM6. (m) PNDI’s molecular structure. (n) *T*_g_ of 80 nm PNDI. (o) *T*_g_ of 40 nm PNDI. All samples were measured at a cooling rate of 0.1 K/s. The corresponding 1st derivative plots are presented in the inset figures for *T*_g_ values verification. For enthalpy overshoot figures, the *T*_g_ is calculated based on the graphical method by equating the two areas as shown in figures of orange and green parts by Moniyhan’s method.

The Flash DSC 2+ is shown in Figure 1a along with a schematic of the ultrathin CP film on top of the heating chamber. The temperature protocol used in this work is shown in Figure 1b. CP thin films with thicknesses ranging from 40 to 116 nm were obtained by spin coating. Keddie and Jones proposed the empirical equation, i.e., 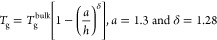 to guide the *T*_g_ depression under confinement after they first reported reduced *T*_g_ values for PS two decades ago.^52^ The bulk state was defined as the film thickness greater than 150 nm since the depressed value of *T*_g_ is only 0.2% relative to *T*_g_^bulk^ (values for each CP can be found in Figure S1) based on empirical equation predictions. Since bulk *T*_g_ varies for different CPs, in this study, we investigated confinement strength, which is defined as the difference between the *T*_g_ of the thin film sample and the *T*_g_ of the bulk sample (*T*_g_–*T*_g_^bulk^). Figure 1c displays the confinement strength as a function of the inverse of film thickness (1/*h*) from 116 to approximately 40 nm, where *T*_g_ was obtained at a cooling rate of 0.1 K/s. The negative values of *T*_g_–*T*_g_^bulk^ were expected since the mobile layer between the polymer and air interface gradually dominated the chain mobility, as evidenced by the reduced *T*_g_ value. Another interesting feature for CPs was observed by comparing the confinement strength of well-studied polystyrene (PS), where the reduction of *T*_g_ of PS was obtained from literature on supported PS thin film.^15^ It is clear that CPs experienced a stronger *T*_g_ depression compared to that of PS, indicating stronger confinement effects. Furthermore, by comparing data points between various CPs, another trend was observed: confinement strength depends on the backbone rigidity of the polymers. The confinement strength increased from flexible polymer to more rigid polymer in the following order: P3HT, PDPPT, PM6, and PNDI. Figure 1d,g,j,m lists the chemical structures of the measured CPs.

Due to the complex dynamics of CPs, it was reported more than one *T*_g_ existed for CPs, including P3HT and PDPPT.^53,54^ The lower *T*_g_ value around room temperature is defined as the *T*_g_ of the mobile amorphous fraction (*T*_*g,*MAF_), and the higher *T*_g_ over 100 °C is defined as the *T*_g_ of the rigid amorphous fraction (*T*_*g,*RAF_). The lower *T*_g_ is associated with the relaxations of the backbone amorphous region, whereas the higher *T*_g_ is constrained by the ordered domains. A similar phenomenon was also observed for PM6; thus, we here used *T*_*g,*MAF_ to discuss the confinement strength, as well as to avoid any additional influence from crystalline domains. The heat flow versus temperatures for various film thickness CP films is displayed in Figure 1e,f,h,i,k,l,n,o, where the arrows indicated the value of *T*_g_. To validate those observed *T*_g_ values, the first derivative of the corresponding heat flow versus temperatures curves can be found in inset figures, where consistent results of *T*_g_ value were obtained. Here, we listed all the *T*_g_ values measured in this study in Table 1 and the other *T*_g_ values of specific thickness can be found in Figure S2.

**Table 1:** Glass Transition Temperatures of Selected CP Thin Films

polymers	thickness (nm)	*T*_*g,*MAF_ (°C)
P3HT	bulk	24.8
P3HT	90	20.9
P3HT	60	18.0
P3HT	40	14.8
PDPPT	bulk	15.4
PDPPT	100	8.0
PDPPT	82	5.4
PDPPT	40	–1.8
PM6	bulk	24.8
PM6	116	18.0
PM6	69	13.0
PM6	55	8.8
PM6	38	5.1
PNDI	bulk	133.0
PNDI	80	119.4
PNDI	40	111.1

**Figure 2:**
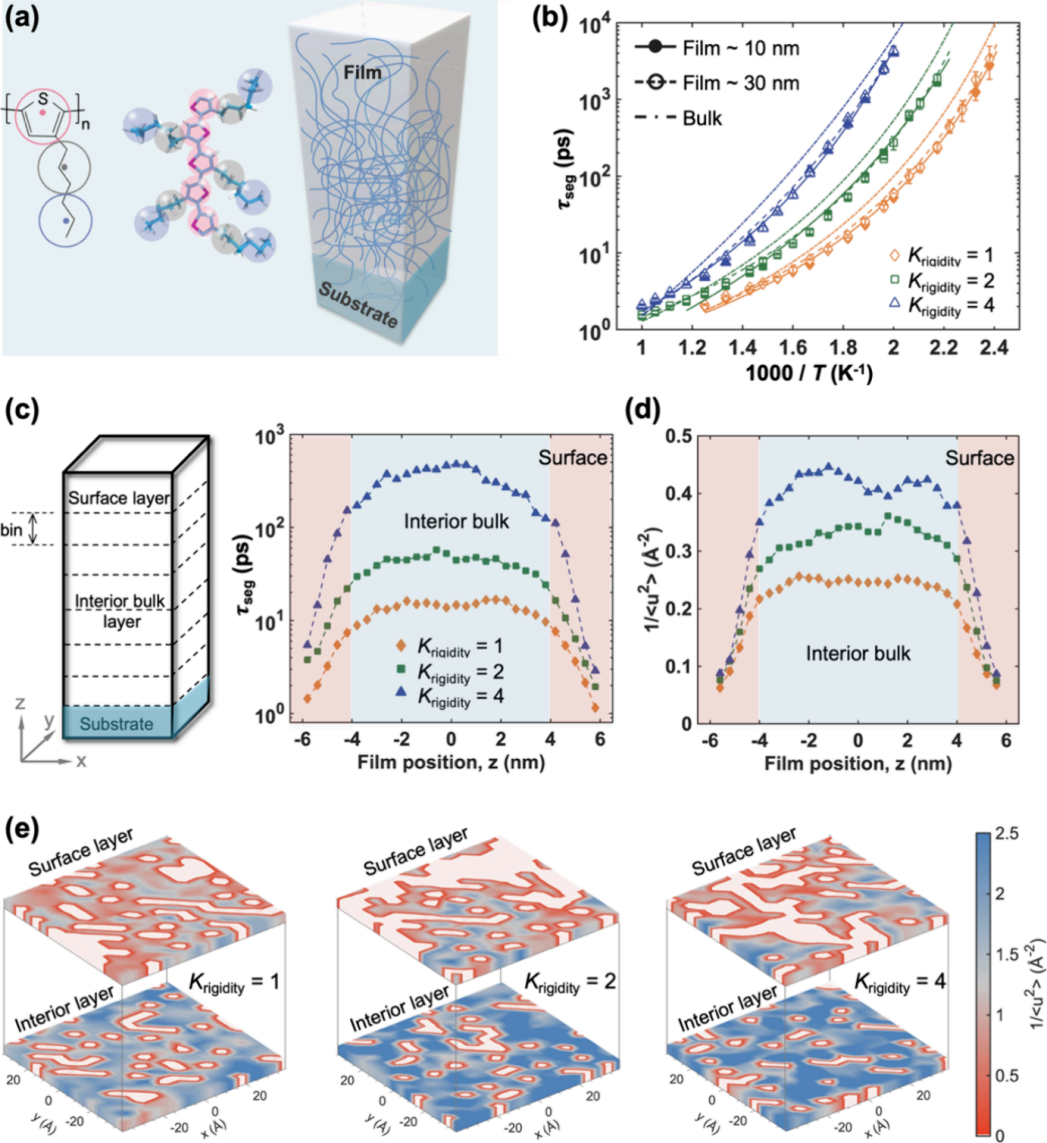
(a) The mapping scheme from all-atomistic model to the three-bead per monomer coarse-grained model for P3HT (left); schematic illustration of polymer thin film (right). (b) *T*-dependent segmental relaxation time τ_seg_ for 10 nm- and 30 nm-thick films comparing with the bulk system at various *K*_rigidity_ values. The solid and dashed curves represent the VFT fits of the τ_seg_ data. (c) The distribution of local τ_seg_ as a function of film position *z* for different *K*_rigidity_ values. (d) Distribution of local molecular stiffness 1/⟨*u*^2^⟩ as a function of film position *z* for different *K*_rigidity_ values. (e) Color maps of the 1/⟨*u*^2^⟩ for the interior layer and surface layer with varying *K*_rigidity_. The red domains correspond to lower local stiffness or higher local mobility, while blue domains correspond to higher local stiffness or lower local mobility. The color map scale is the same for all images (color online).

In addition to experimental investigations by DSC, coarse-grained molecular dynamics (CG-MD) simulations were conducted to enhance our understanding of the interplay between confinement strength and backbone rigidity. A chemistry-specific CG model, informed by the all-atomistic (AA) model of P3HT (Figure 2a), was employed. In this model, each P3HT monomer was characterized by three distinctive CG beads—P1, P2, and P3—corresponding to the thiophene ring, the first three consecutive hexyl side chain methyl groups, and the last three hexyl side chain methyl groups, respectively. The CG model description was discussed in detail in the Experimental Section. In our modeling, we applied bending constraints to three consecutive beads along the polymer chain to govern its bending behavior. To modulate the bending rigidity, a rescaling factor, *K*_rigidity_, was introduced for angular stiffness, providing control over polymer backbone rigidity. Specifically, a *K*_rigidity_ value of 1 aligns with the CG model representing P3HT. In contrast, increasing the *K*_rigidity_ value to a higher value allows us to simulate more rigid backbones, such as donor–acceptor CPs used in this study. The chain rigidity of CPs can be quantified by *L*_p_, representing the characteristic length for the decay of correlations in the backbone tangents. We systematically explored the influence of rigidity on the dynamic and mechanical behaviors of CPs. Our findings, illustrated in Figure S3, demonstrated an increase in *L*_p_ as *K*_rigidity_ rises. This observation supports the efficacy of using *K*_rigidity_ to control the polymer chain rigidity.

We began by analyzing the segmental structural relaxation time τ_seg_, a pivotal dynamic quantity crucial for understanding glass formation dynamics, particularly the *T*_g_. We first investigated the influence of *K*_rigidity_ on τ_seg_ in the bulk state, and the *T*-dependent variations of τ_seg_ are presented in Figure S4. Notably, τ_seg_ showed a substantial increase upon cooling at a fixed *K*_rigidity_. The progressive introduction of *K*_rigidity_ led to considerably longer τ_seg_ values at lower temperatures, while the impact of changing *K*_rigidity_ on τ_seg_ was relatively weak at higher temperatures, which aligned with the empirical trend observed in linear polymer melts.^36,55,56^ The non-Arrhenius behavior of τ_seg_ at low temperatures is well-described by the Vogel–Fulcher–Tammann (VFT) expression,^57^ τ_seg_(*T*) = τ_0_ exp [*DT*_0_/(*T* – *T*_0_)], where τ_0_, *D*, and *T*_0_ are fitting parameters characterizing the glass formation relaxation process. Specifically, the Volgel temperature *T*_0_ marks the “end” of glass formation, where τ_seg_ formally extrapolates to an infinite value. *D* is inversely related to the fragility parameter *K* (i.e., *K* ≡ 1/*D*).^58^ Based on the VFT fit, *T*_g_ was estimated by extrapolating τ_seg_ data to the empirical observation time scale. To minimize uncertainty in this long extrapolation, we employed a “computational” *T*_g_ criterion, τ_seg_(*T*_g_) = 1 ns, analogous to the experimental convention of defining *T*_g_ (i.e., τ_seg_(*T*_g_) = 100 s).^59,60^ There is a linear correlation between *T*_g_ estimated using this computational criterion and *T*_g_ determined by the traditional experimental method, as noted in previous studies.^59,61^

Following this, we conducted comparisons between thin films and bulk systems, focusing on the *K*_rigidity_-dependent depression of thin film *T*_g_. Figure 2b shows a comparison of τ_seg_ for bulk systems and 10 and 30 nm-thick films at various *K*_rigidity_ values as a function of temperature. Notably, τ_seg_ was consistently lower in the thin film compared to that in the bulk system, indicating that the free surface induced higher mobility of polymer chains across different chain rigidity cases. Moreover, films with higher *K*_rigidity_ exhibited a more substantial deviation of τ_seg_ from the bulk, suggesting that the free-surface effect on film τ_seg_ was more pronounced for higher *K*_rigidity_. Additionally, Table S1 summarizes the corresponding *T*_g_ values, where we defined the difference between the film and bulk *T*_g_ as Δ*T*_g_ = *T*_g_^bulk^ – *T*_g_^film^. Notably, we observe that Δ*T*_g_ decreased as the film thickness increased (i.e., from 10 to 30 nm), which could be attributed to the diminishing proportion of the free-surface layer in thicker films, aligning with our experimental observations. However, irrespective of film thickness, the free-surface-induced decrease in *T*_g_ was more prominent for polymer systems with higher *K*_rigidity_, qualitatively agreeing with our experimental findings illustrated in Figure 1c. It is worth mentioning that Vogt et al.^62^ investigated the dependence of backbone rigidity on molecular dynamics, where the flexible backbone was subjected to more pronounced confinement effects. Here, we attributed such a discrepancy to the different molecular architectures. The persistence length in our system is much higher, almost as large as 1 order of magnitude.

To further interpret our results and quantify the length scale of the perturbation at the free surface, we examined the distribution of local relaxation associated with segmental mobility within the film. Figure 2c illustrates the spatial distribution of local segmental relaxation time τ_seg_ as a function of film position *z* for various *K*_rigidity_ values. Notably, τ_seg_ approaches near-zero values at the free-surface boundary and increases almost linearly with depth in the film. About 2 nm away from the free surface, local τ_seg_ saturated to values resembling bulk-like behavior in the interior region. Films with higher chain rigidity exhibited higher τ_seg_ in the interior bulk region, while there was a minimal difference in τ_seg_ for different *K*_rigidity_ values near the free surface. In these simulations, varying the rigidity allowed us to mimic systems ranging from highly rigid (e.g., PNDI) to flexible (e.g., P3HT) polymers. Higher rigidity polymers exhibited more pronounced differences in segmental relaxation times between the surface and interior bulk, whereas flexible polymers adapted more easily to confinement. These findings emphasize the significant role of polymer rigidity in determining the effects of confinement, aligning well with experimental observations. Furthermore, we analyzed the local stiffness 1/⟨*u*^2^⟩ of polymer chains by calculating the mean-square displacement (MSD) along the film height as shown in Figure 2d, where higher 1/⟨*u*^2^⟩ indicates lower particle mobility. Analogous to the τ_seg_ pattern, the local stiffness experiences a parallel increase and convergence within the interior region, and the soft surface layer was estimated to be approximately 2 nm, aligning with findings from prior simulation studies.^63,64^ The impact of chain rigidity on mobility is more pronounced in the interior region, exhibiting a minimal influence near the free surface. The detailed simulation results can be found in Figure S5. We also investigated the influence of interaction strength ε_sp_ on mobility and found that weaker substrate cohesion results in a clear mobility gradient near the substrate, which disappears with stronger cohesion, leading to uniform mobility across the film (Figure S6). For enhanced comparison, 3D color maps of local 1/⟨*u*^2^⟩ are depicted in Figure 2e. As *K*_rigidity_ increased, the interior region displayed a higher degree of dynamical heterogeneity, evident in the heterogeneous distribution of local 1/⟨*u*^2^⟩, while the surface layer exhibited minimal variation in terms of 1/⟨*u*^2^⟩. This discrepancy elucidated the larger *T*_g_ depression for thin films with a higher *K*_rigidity_.

### Confinement Effect on Film Morphology and Mechanical Properties

**Figure 3:**
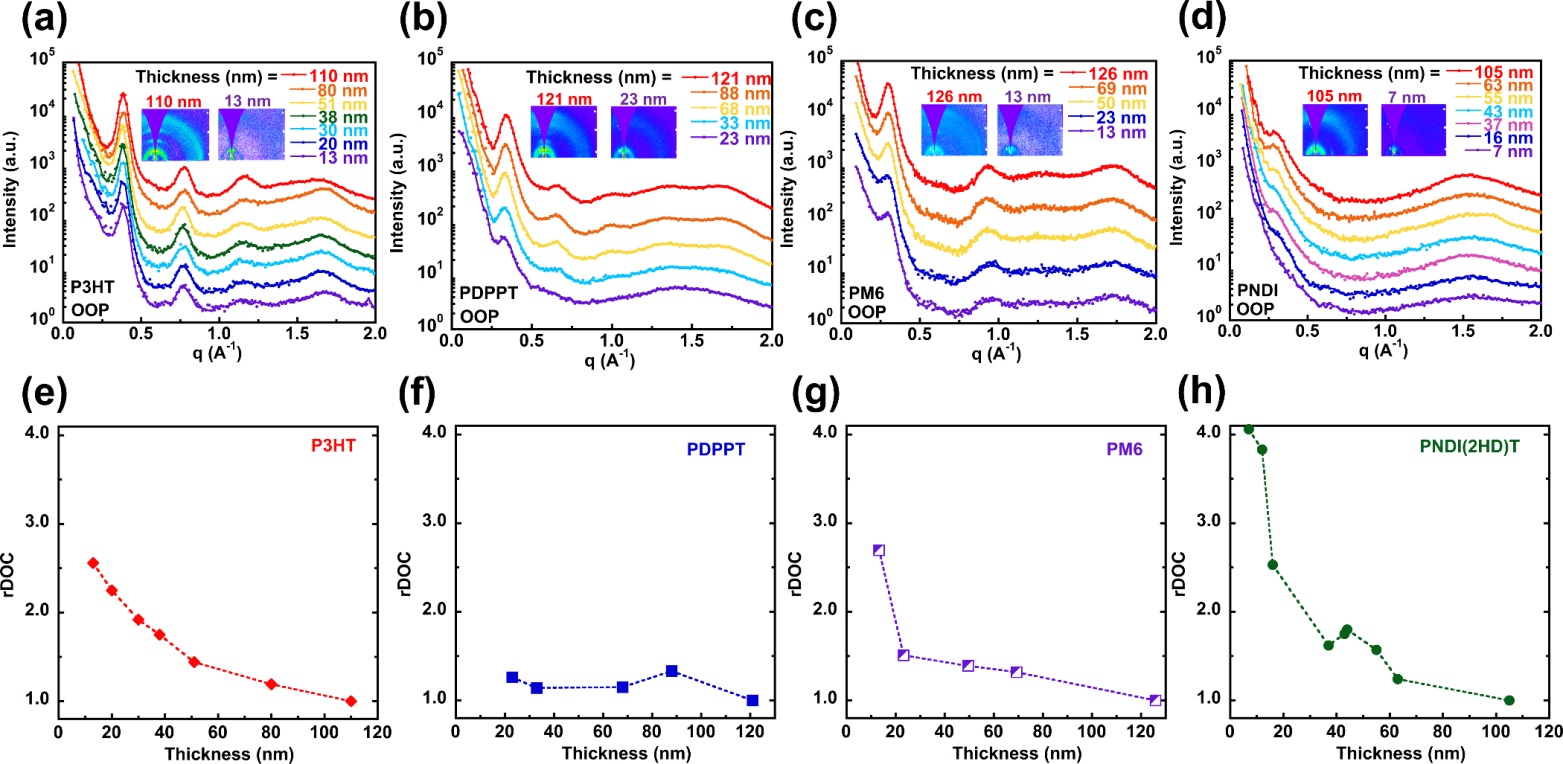
Representative out of plane GIWAXS 1D plots for (a) P3HT, (b) PDPPT, (c) PM6, and (d) PNDI; relative degree of crystallinity plotted against film thickness for (e) P3HT, (f) PDPPT (g) PM6, and (h) PNDI. The inset figures are the representative GIWAXS 2D images for the highest and lowest film thickness. The rDOC values are normalized to film thickness.

The morphology of the CPs with different film thicknesses was studied by grazing-incidence wide-angle X-ray scattering (GIWAXS). The 2D scattering patterns of the samples with the highest and lowest film thickness are shown in the inset figures of Figure 3a–d, accompanied by the corresponding 1D scattering profiles (intensity *I* vs scattering vector *q* plot) for P3HT (Figure 3a), PDPPT (Figure 3b), PM6 (Figure 3c), and PNDI (Figure 3d). The 2D scattering patterns for other film thicknesses can be found in Figures S7–S10.

The CP’s physical and optoelectronic properties are influenced by the degree of crystallinity. One can measure the degree of crystallinity of a given polymer by DSC, density measurements, infrared spectroscopy, and X-ray scattering.^65^ Since our samples are thin films, GIWAXS is the only feasible way to obtain the relative degree of crystallinity (rDOC). The rDOC of the CP thin films was then calculated based on the pole figure of (100) peaks, where the corresponding pole figures are illustrated in Figure S11. The rDOCs for all of the CPs are organized in Figure 3e–h, normalized by the thickest sample value as rDOC = 1. For P3HT thin films shown in Figure 3e, rDOC increased monotonically as the film thickness decreased. The rDOC for the thinnest film was doubled that of the thickest film. For PM6 thin films, the rDOC increased by 50% from over 100 to 20 nm and further increased by 120% as film thickness dropped to 10 nm. A similar trend was observed for DPP thin films, where the rDOC increased by 30% from 120 to 20 nm. Lastly, PNDI experienced the largest enhancement of crystallinity for the thinnest film, which showed an rDOC as high as four times that of films over 100 nm (Figure 3h). The rDOC measurements were done at room temperature, where P3HT, with a subroom temperature *T*_g_, exhibited higher chain mobility, allowing the chains to pack well through chain relaxation from low *T*_g_ side chain structures. Therefore, the rDOC increased as the chain became more mobile at reduced thickness. In contrast, the most rigid PNDI CP has the least chain mobility at room temperature while experiencing the highest confinement strength. In the ultrathin film state, the significantly enhanced chain mobility resulting from the highest *T*_g_ depressions facilitated the chain packings, leading to a higher degree of crystallinity upon confinement. PDPPT and PM6 have intermediate chain rigidity and confinement strength; therefore, the trends in rDOC changes followed accordingly. Since the morphology and rDOC apparently influence the mechanical and electronic properties, we explored the confinement effects upon mechanical strength and carrier charge mobility in the following discussions.

**Figure 4:**
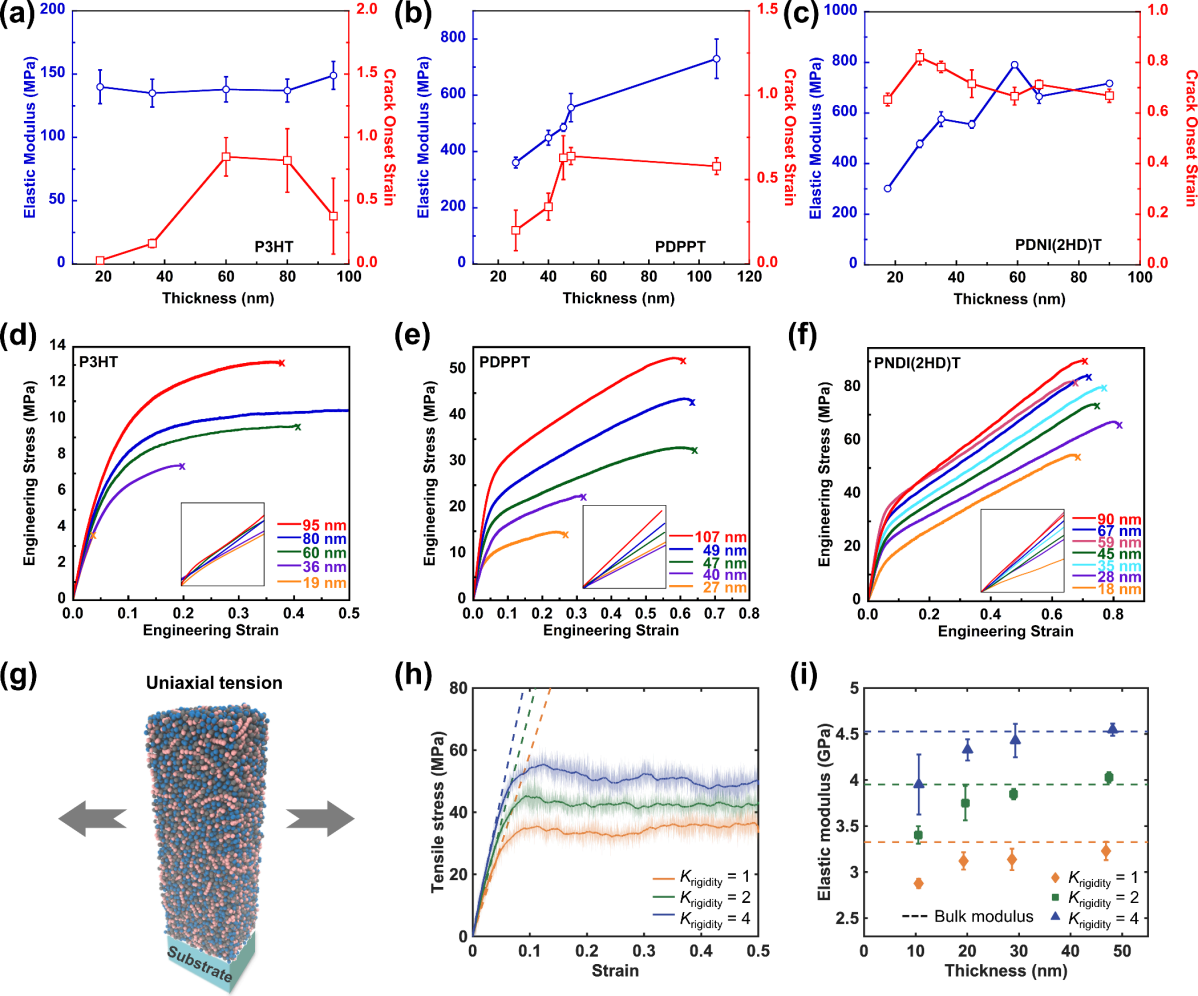
Mechanical properties of selected CPs: elastic stress and COS versus film thickness for (a) P3HT, (b) PDPPT, and (c) PNDI; engineering stress as a function of engineering strain for various film thickness for (d) P3HT, (e) PDPPT, and (f) PNDI, where the inset figures are the linear regions of the stress–strain curves. (g–i) Simulation result. (g) Schematic illustration of uniaxial tension of the polymer thin film. (h) Stress–strain response for thin film with a thickness ∼20 nm for different chain rigidity *K*_rigidity_. The elastic modulus is extracted from the slope shown by the dashed lines. (i) Thickness dependence of the elastic modulus of thin film for different *K*_rigidity_ values. The dashed lines indicate the corresponding modulus in the bulk state. Panels (a) and (d) are adapted with permission from ref (66). Copyright 2018, John Wiley and Sons.

Next, we studied the mechanical properties of confined CP thin films using a unique pseudo free-standing tensile tester. To investigate the influence of chain rigidity on mechanical properties, P3HT, PDPPT, and PNDI polymers were tested here due to the distinct *L*_p_ values. The elastic modulus (*E*) and crack onset strain (COS) were plotted against the film thickness for P3HT, PDPPT, and PNDI, as shown in Figure 4a–c. The engineering stress (σ)–engineering strain (ε) curves for each individual sample are summarized in Figure 4d–f. The mechanical properties of thickness-varying P3HT were adopted from our group’s previous work published by Zhang et al.^66^ The *E* of P3HT was independent of film thickness within errors, where COS reached its highest value in moderately thin films but was reduced to the lowest number for the thinnest sample. However, for DPP CPs, the *E* dropped monotonically within decreasing film thickness (730 to 360 MPa), whereas the COS first remained constant at 50% between 100 and 50 nm and then gradually dropped to 20% from 50 down to 27 nm. A similar trend was also observed for NDI CPs, where the *E* was reduced from 720 to 300 MPa at a film thickness of 18 nm and the COS was maintained until 30 nm thickness. We attributed the decreased stretchability to the loss of entanglement upon confinement as well as changes in the rDOC of CP thin films.

CG-MD simulations were again conducted to further characterize the mechanical response of thin films through uniaxial tension along the *x*-direction. We measured the *E* of thin films with varying chain rigidity *K*_rigidity_ and film thicknesses. Figure 4g illustrates the MD simulations of tensile tests, and the typical stress–strain curves for thin films with different *K*_rigidity_ values are presented in Figure 4h. These stress–strain responses for thin films exhibited similarities to the bulk behavior shown in Figures S12 and S13. Moreover, the thickness dependence of *E* for thin films with different *K*_rigidity_ values is depicted in Figure 4i. The *E* increased with film thickness, gradually converging to its modulus in the bulk state for each *K*_rigidity_. Notably, the film with a higher *K*_rigidity_ exhibited a greater *E* value over different thicknesses, attributed to the free-surface-induced reduction in the film modulus due to the enhanced mobile layer at the free surface, as discussed earlier. These results emphasize how higher rigidity strengthens the mechanical response across thicknesses, consistent with observed trends in free-surface effects.

### Effect of Confinement On Electronical Property

**Figure 5:**
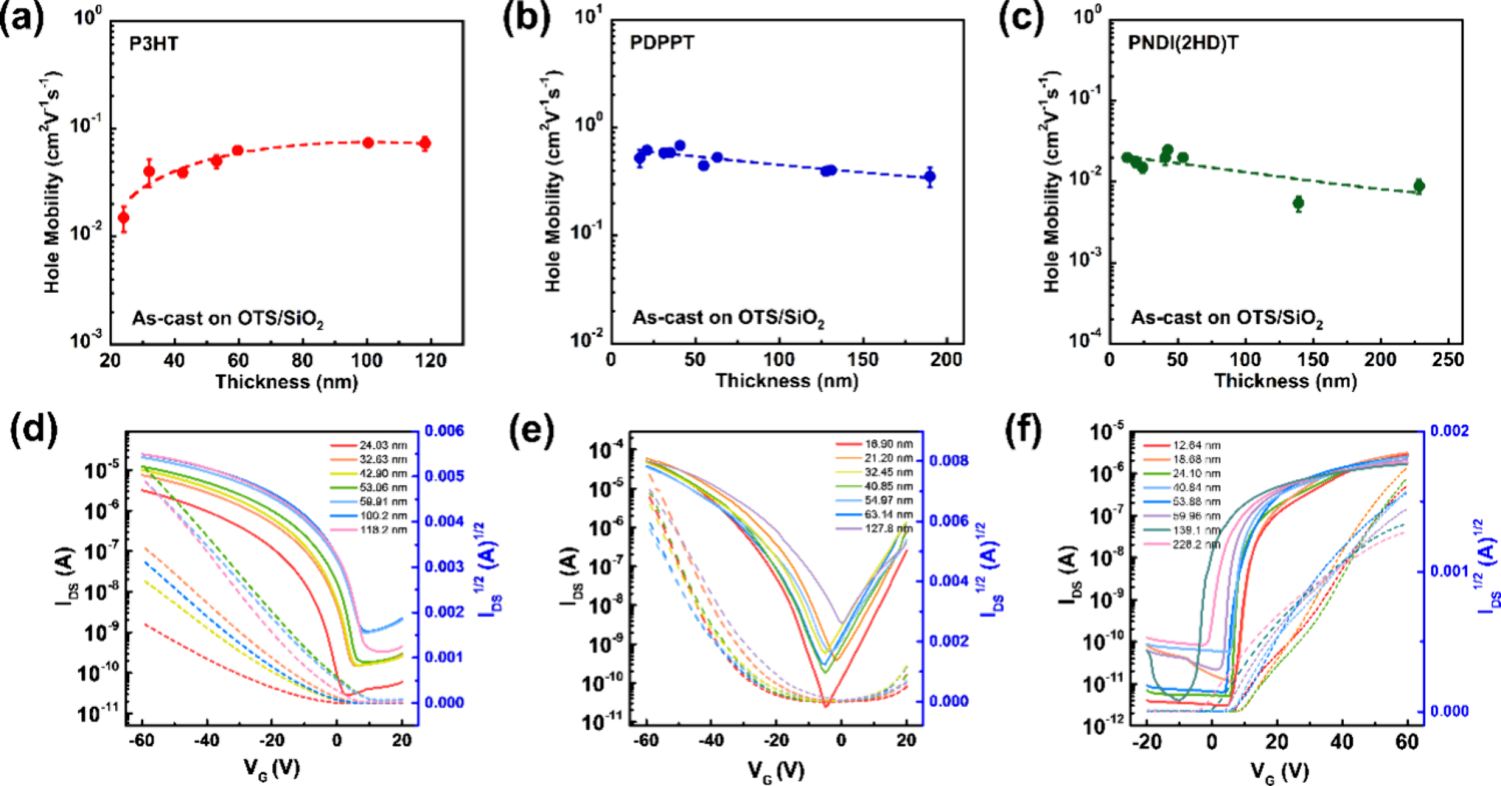
Charge carrier mobilities vs film thickness plot for (a) P3HT, (b) PDPPT, and (c) PNDI; corresponding transfer curves for (d) P3HT, (e) PDPPT, and (f) PNDI.

The confinement effects upon the thin film device’s electronic properties are one of the scopes of this project, especially their impact on the charge carrier mobilities. To quantitatively investigate the influence of varying film thickness on charge carrier mobilities, bottom-gate top-contact field-effect transistors were measured. Figure 5a reports the relationship between charge carrier mobility and film thickness for P3HT on an octyltrimethoxysilane (OTS)-treated doped silicon wafer with 300 nm silicon oxides as the dielectric layer. The thickest P3HT film showed a charge carrier mobility of 0.07 cm^2^V^–1^s^–1^ consistent with literature-reported values.^67^ The charge carrier mobility is weakly dependent on the film thickness. A value of 0.02 cm^2^ V^–1^ s^–1^ was obtained for the thinnest film of P3HT. Despite the descending dependence of charge mobility on film thickness reported previously by the literature,^67,68^ our results indicated that no significant charge mobility loss was found as film thickness was reduced, similar to another report in the literature.^69^Figure 5d shows the transfer curve of P3HT at various film thicknesses. The threshold voltage (*V*_th_) ranged from −1 to −17 V. The on/off current ratio was independent of film thickness and maintained at approximately 1 × 10^4^, indicating high performance for such a transistor. The mobility of the p-type PDPPT was characterized and is plotted in Figure 5b, where the charge mobilities for the largest and smallest thickness films were 0.4 and 0.6 cm^2^ V^–1^ s^–1^, respectively. Over a wide range of 200 nm, the charge carrier mobility was independent of film thickness. The transfer curve shown in Figure 5e, on the other hand, displayed opposite trends compared with P3HT. The on/off ratio decreased within a smaller film thickness, while *V*_th_ remained almost unchanged. Finally, the n-type polymer PNDI exhibited an average charge mobility of 0.017 ± 0.006 cm^2^ V^–1^ s^–1^ ranging from a film thickness of 12 to 228 nm film thickness. Again, the film thickness played a minor role in the charge carrier mobilities, as shown in Figure 5c. Although the on/off ratio (∼10^5^) was not influenced by film thickness, *V*_th_ increased for the smallest thickness film, as shown in the transfer curve in Figure 5f. The forward and backward transfer curves maintained traces in the hysteresis measurement, as shown in Figure S14, indicating no trapping effects on the low energy surface of the OTS-modified dielectrics during gate voltage scanning. In other words, the extracted mobilities originated from the semiconductors with different thicknesses themselves and are not affected by the semiconductor/dielectric interface. In general, the thickness-dependent OFET measurements confirmed that all three CPs maintained relatively high charge mobilities even at very thin film thickness down to 10 nm or so. These findings demonstrated that charge carrier transport in an OFET device primarily occurs in the first layer of the semiconductor at the dielectric interface, as reported in the literature.^70^ It is worth noting that the results of V_th_ for the devices with OTS-modified SiO_2_ as dielectrics, in Figure 5d,e obviously tend to turn on earlier with the increase of semiconductor thickness, indicating the numerous trapping sites created in thicker semiconducting polymers benefits reducing onset gate voltage.^71−73^

## Discussions

**Figure 6:**
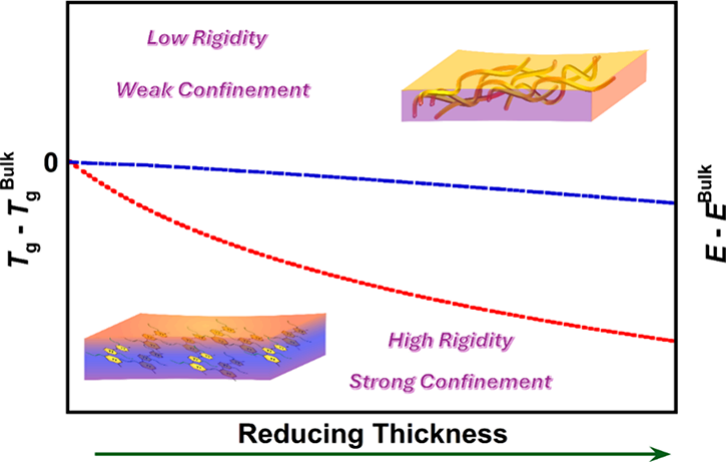
Schematic plot of the influence of chain rigidity on confinement strength.

Here, we discuss the correlations and variations in the effects of nanoconfinement on traditional polymers and conjugated polymers using a schematic plot in Figure 6. There has been intensive work regarding polymer glasses in confinement; however, the investigated subjects are mostly fully amorphous polymers, such as PS or PMMA. One of the reasons is their simple molecular structure, which is suitable for fundamental molecular mobility studies since confinement will only influence the backbone amorphous regions. On the other hand, CP has a heterogeneous system including the highly rigid backbone with several stiffer building blocks and the highly flexible alkyl side chains expanding the free volume. Besides the mismatch between backbone and side chain mobilities, most CPs are semicrystalline, so confinement not only influences amorphous regions but also alters small crystals/paracrystalline fractions, resulting in complex interfaces between amorphous/crystalline fractions. Therefore, the confinement strength shows different behaviors between those of traditional amorphous polymers and conjugated semicrystalline polymers.

In addition to confinement strength resulting from different film thicknesses, we can also examine it using various cooling rate experiments. It has been reported a cooling rate dependence of *T*_g_ depressions by various groups, where the amount of reduced *T*_g_ of ultrathin film would be compensated by faster cooling rates.^74−76^ For PS, the thinner film will show a lower *T*_g_ value, but *T*_g_ is a kinetic process, and the faster cooling rates of 1000 K/s will lead to a higher value. Thus, the overall changes for *T*_g_ depression would be minimal at higher cooling rates and it will gradually become appreciable as cooling rates are reduced.^76^ On the contrary of PS, PDPPT showed a depressed *T*_g_ value even when it was cooled at the much faster cooling rate of 1000 K/s (as shown in Figure S15), indicating the stronger confinement strength of the CP thin film. Another interesting feature between traditional polymers and CPs is their influences on crystallization behaviors caused by side chain structures, especially on the degree of crystallinity under confinement. As flexible polymers are trapped in confined environments, the surrounding wall significantly reduces the mobilities of tethered crystalline chains and slows down the crystallization, resulting in smaller rDOC relative to the bulk.^77−79^ On the other hand, our measurements showed consistent results for all of these four CPs under confinement; the rDOC was clearly enhanced as film thickness continuously decreased. We may attribute this discrepancy to the stiff/rigid backbone with strong intermolecular forces to increase the tendency for chain aggregation, which provides a much faster crystallization rate. Besides that, the strong confinement effects observed for CP polymers can enhance the free surface effects to accelerate the crystallization rate under a confined environment. Tuning the crystallinity for CP is beneficial for device engineering since the charge transport mobility is positively associated with increased rDOC.

The confinement strength also influences the mechanical properties of the thin film. For similar film thickness nonconjugated polymers vs CPs, the amount of reduced *T*_g_ will determine the state of the materials. For example, ultrathin PS film tested at room temperature showed glassy behaviors, whereas PDPPT was in a viscoelastic state under the same conditions. Therefore, we would expect different mechanical responses. The elastic modulus dependence on strain rate was weak for PS since it was stretched in a glassy state, where the frozen chains did not have enough degree of freedom to adjust to the applied deformations. However, the gained mobilities from decreased *T*_g_ for viscoelastic PDPPT was more sensitive to deformation rate resulting in a high elastic modulus at a fast strain rate.^66^ Energy loss test during cyclic loading–unloading process, known as hysteresis effects, is another interesting aspect comparing nonconjugated and conjugated polymers. For the PS thin film, the unloading curves followed the previous loading trace, indicating elastic behavior once the applied stress was removed. However, for PDPPTVT thin film, the loading and unloading curves were not overlapping, indicating that both elastic and viscoelastic behaviors existed as more mobilities were retrieved under stronger confinement effects.^66^ Furthermore, stress relaxation is associated with chain mobilities, so we would expect much more stress relaxation to occur for more strongly confined materials, which needs to be addressed while preparing stretchable organic electronic devices.

The dependence of elastic modulus upon film thickness could also behave differently between traditional flexible polymers and semirigid CPs. Previous work conducted by Saito et al.^80−82^ showed increased elastic modulus as film thickness gradually decreased for block copolymers. They explained that findings using two-layer models that the top surface was dominated by high modulus PS domains and such PS-rich layer thickness kept constant as the total film thickness was reduced, resulting in more appreciable high modulus film. For our CP system, each CP is a homopolymer, so the elastic modulus decreased as the film thickness decreased, owing to the surface mobile layer. Additionally, the influence of rDOC on elastic modulus was intensively investigated for bulk nonconjugated polymers.^83,84^ In general, the modulus is greatly affected by the state of orientations of the crystalline phases, whereas the degree of crystallinity showed only less obvious effects. As the rDOC increases for polyesters, the decreasing amorphous region could contribute to higher elastic modulus due to the reduced free volume.^85,86^ Furthermore, the more ordered and well-packed structure could lead to stronger intermolecular interactions, resulting in enhanced elastic modulus. Nonetheless, for our confined CP systems, we have two competing effects regarding the elastic modulus: the increased rDOC vs the enhanced dynamics from the mobile layer. Our results showed that enhanced mobilities dominated the influence on elastic modulus at a very thin layer, so we observed a reduced elastic modulus even though the rDOC was relatively higher compared to the bulk film.

## Conclusions

In summary, representative high-performance CPs were adopted to investigate the influence of the film thickness on their physical and electronic properties. The chain mobilities of selected CPs, ranging from the most flexible P3HT to the most rigid PNDI, were examined using Flash DSC. The chain mobility, indicated by *T*_g_, was found to decrease as film thickness was reduced due to free surface effects. The confinement strength, defined as the change in the *T*_g_ value between bulk and thin film samples, was associated with persistence length. Molecular simulations confirmed our hypothesis that more rigid CPs are subject to stronger confinement strength as film thickness decreases. The relative crystallinity of these CPs was explored by GIWAXS measurements, which showed that all CPs exhibited enhanced rDOC at the smallest film thickness. The mechanical and electronic properties under confinement were also examined. The elastic modulus decreased as film thickness was reduced, with PDPPT and PNDI showing significantly reduced elastic modulus. However, the carrier charge mobilities were independent of film thickness, indicating that good charge mobility was maintained even at relatively smaller film thicknesses. The comprehensive understanding of confinement effects upon conjugated polymer properties will be beneficial to the design of portable/flexible organic electronics in the future.

## Supporting Information Available

Glass transition temperature measurements for various film thickness CP, simulation results regarding molecular mobilities under confinement, GIWAXS 2D images under confinement, pole figures for rDOC calculations, and hysteresis curve for charge mobilities for various film thickness CP (PDF)
