# Supplementary material for: Unveiling Strong Thin Film Confinement Effects on Semirigid Conjugated Polymers
Source: Macromolecules. 2024 Sep 23;57(19):9121–34. doi: 10.1021/acs.macromol.4c01500 (PMC11468787; doi:10.1021/acs.macromol.4c01500)
Supplement: Supplementary file 1 — ma4c01500_si_001.pdf [file ma4c01500_si_001.pdf]

Supporting Information

Unveiling Strong Thin Film Confinement Effects on Semi-rigid Conjugated Polymers

Haoyu Zhao[a], Zhaofan Li[b], Yunfei Wang[a], Qi-An Hong[c], Wenjie Xia[b*], Yu-Cheng Chiu[c*], and Xiaodan Gu[a*]

[a]School of Polymer Science and Engineering, The University of Southern Mississippi, 118 College Drive, Hattiesburg, MS 39406, United States of America

[b]Department of Aerospace Engineering, Iowa State University, 2433 Union Dr, Ames, IA 50011, United States of America

[c]Department of Chemical Engineering, National Taiwan University of Science and Technology, Taipei City, 10607, Taiwan

*E-mail: wxia@iastate.edu, ycchiu@mail.ntust.edu.tw, xiaodan.gu@usm.edu

This file includes:

Figure S1 to S15

Table S1

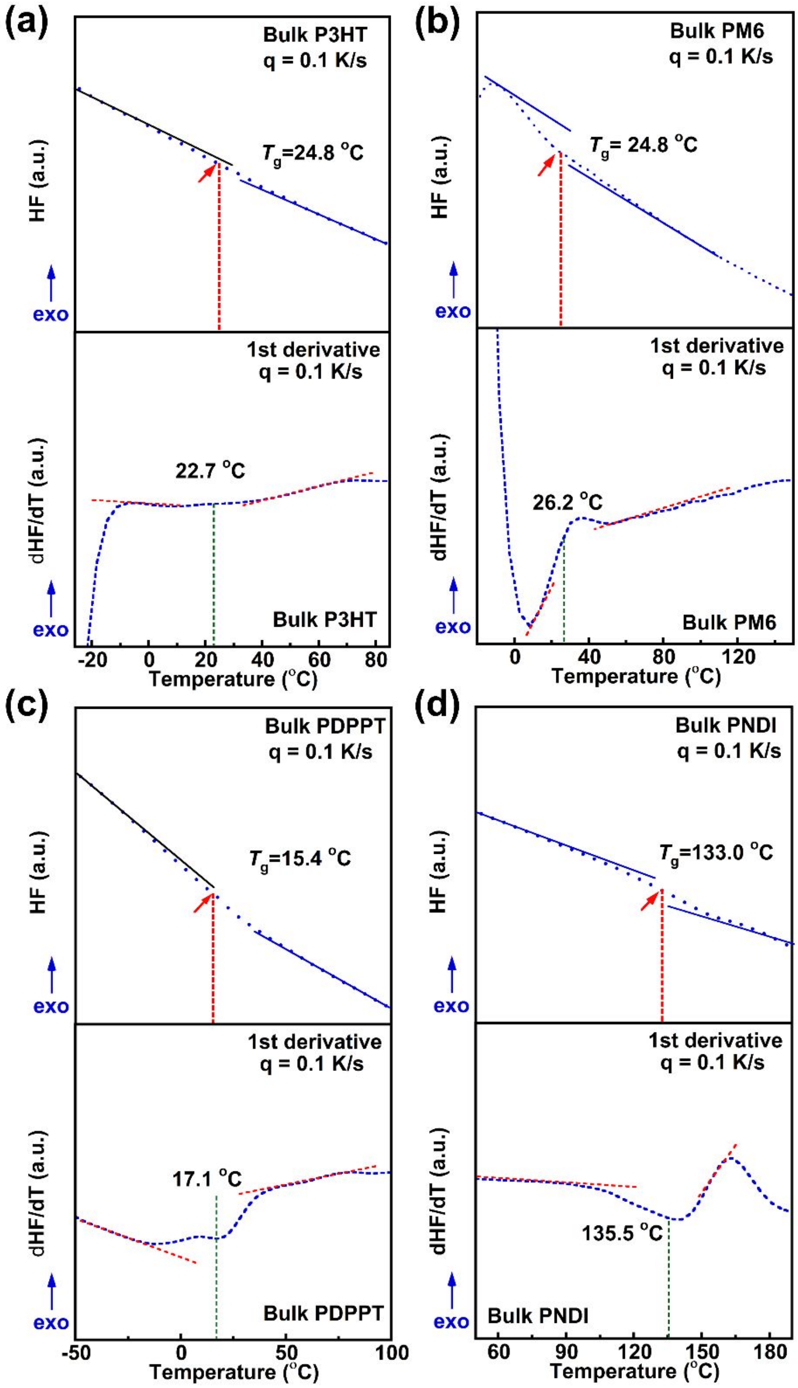

Figure S1. Flash DSC heat flow vs. temperature for bulk CPs T_g measurements **a).** top: bulk T_g of P3HT at cooling rates of 0.1 K/s; bottom: corresponding 1*st* derivative plots for verification **b).** top: bulk T_g of PDPPT at cooling rates of 0.1 K/s; bottom: corresponding 1st derivative plots for verification **c).** top: bulk T_g of PM6 at cooling rates of 0.1 K/s; bottom: corresponding 1st derivative plots for verification **d).** top: bulk T_g of PNDI at cooling rates of 0.1 K/s; bottom: corresponding 1st derivative plots for verification where dashed lines represent glassy and liquid lines and the arrow indicates the occurrence of glass transition temperatures.

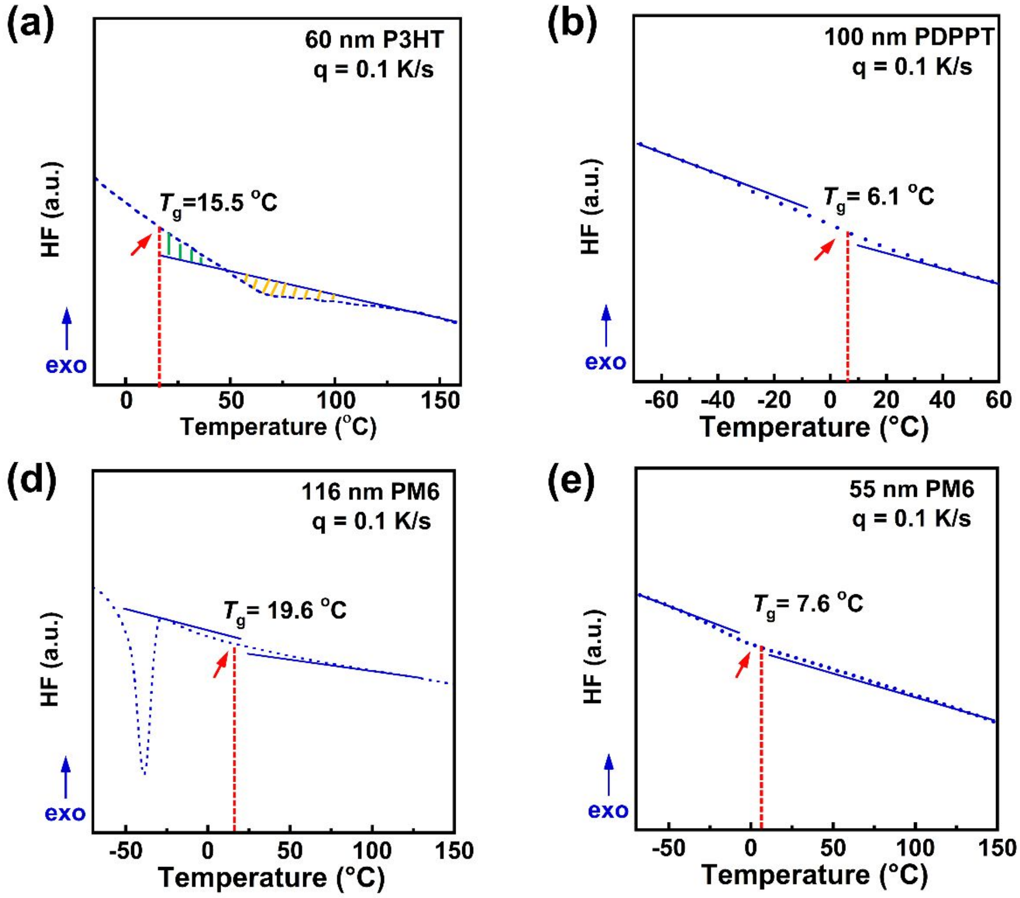

Figure S2. Flash DSC heat flow vs. temperature for CPs T_g measurements at specific film thickness **a)** 60 nm P3HT* **b)** 100 nm PDPPT **c)** 116 nm PM6 **d)** 55 nm PM6, where dashed lines represent glassy and liquid lines and the arrow indicates the occurrence of glass transition temperatures.

*For enthalpy overshoot appeared figures, the T_g is calculated based on the graphical method by equating the two areas as shown in figures of orange and green parts, reported by Moniyhan's method

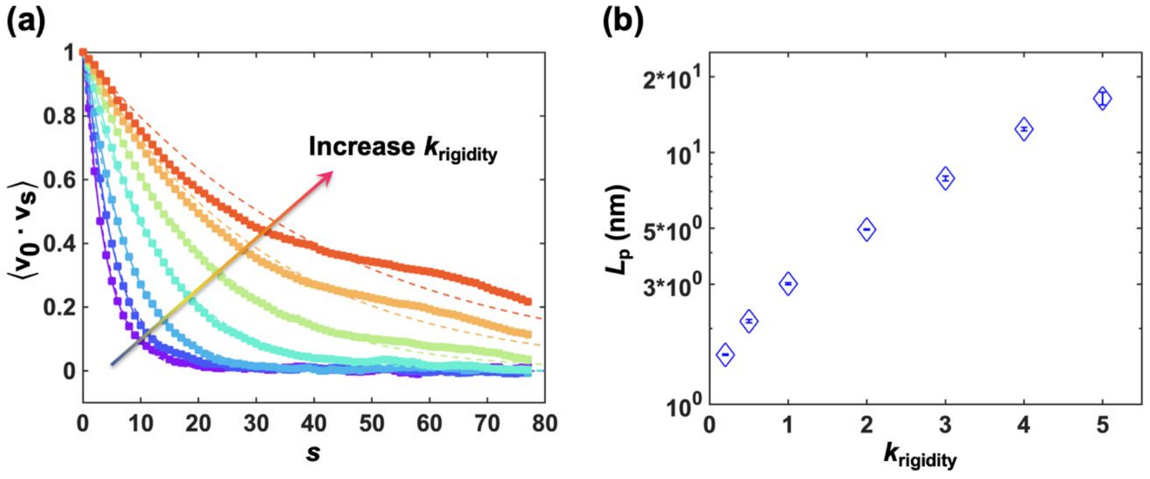

Figure S3. Persistence length L_p calculation in MD simulations. We performed the L_p calculation by considering the beads along the backbone chain contour. $\mathbf{v}_s$ was denoted as the tangent vector connecting two segments s and s+1, and the L_p was determined from the tangent-tangent correlation function $\langle \mathbf{v}_0 \cdot \mathbf{v}_s \rangle$ with the average taken over all trajectories, $\langle \mathbf{v}_0 \cdot \mathbf{v}_s \rangle = e^{-s/n_p}$, where n_p is the persistence length measured in the number of monomers. L_p is taken as $l_0 \cdot n_p$, in which l_0 is average monomer repeat unit length. **a).** Tangent-tangent correlation function with increasing the $K_{rigidity}$. **b).** L_p as a function of $K_{rigidity}$.

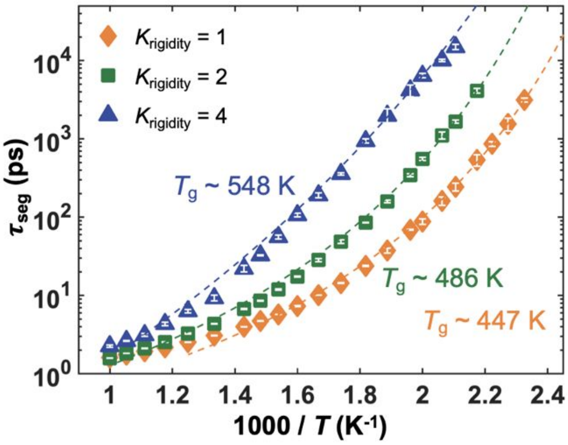

Figure S4. T-dependent segmental relaxation time τ_{seg} for bulk states with different $K_{rigidity}$ values. The dashed curves represent the fits of τ_{seg} data using the VFT equation, allowing for the estimation of the glass transition temperature T_g.

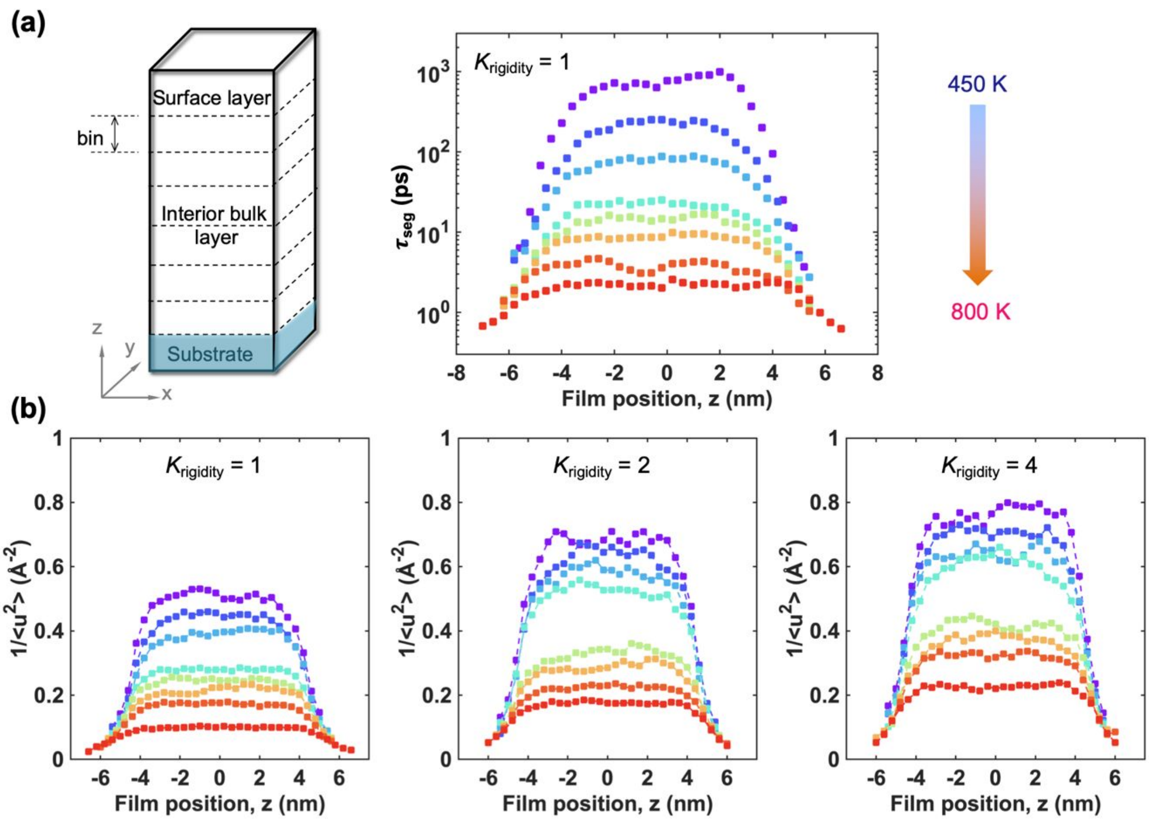

Figure S5. a). The spatial distribution of local τ_{seg} as a function of film position z with K_{rigidity} of 1 for different temperatures. **b).** The distribution of local molecular stiffness $1/\langle u^2\rangle$ as a function of film position z for different K_{rigidity} under varying temperatures.

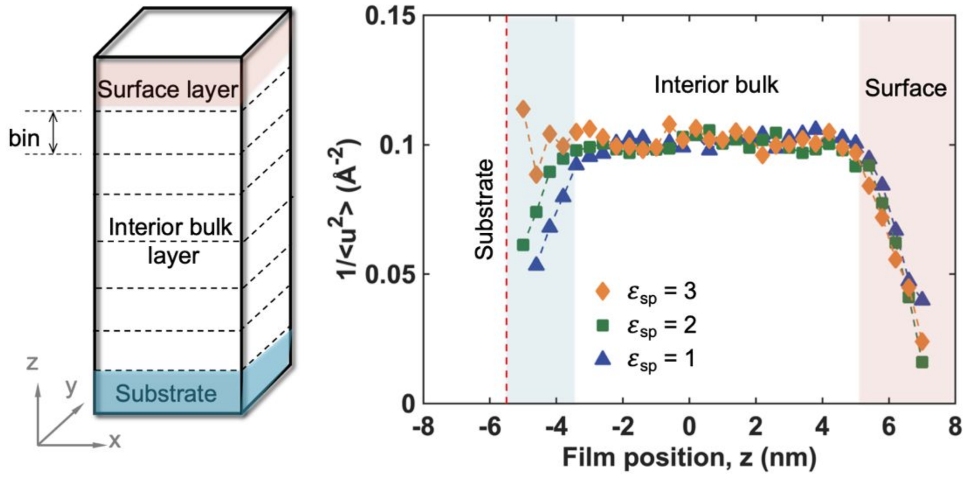

Figure S6. Schematic of supported polymer thin film consisting of substrate, interior bulk-like layer, and softer surface layer. Spatial distribution of the local stiffness, $1/\langle u^2\rangle$, as a function of film position z from the substrate−film interface to the free surface. $\varepsilon_{\mathrm{sp}}$ indicates the energy strength of attraction to the substrate, which is varied from 1 to 3 kcal/mol in simulations. In the special case where $\varepsilon_{\mathrm{sp}} = 0$ kcal/mol, the system becomes a free-standing thin film with no substrate interaction.

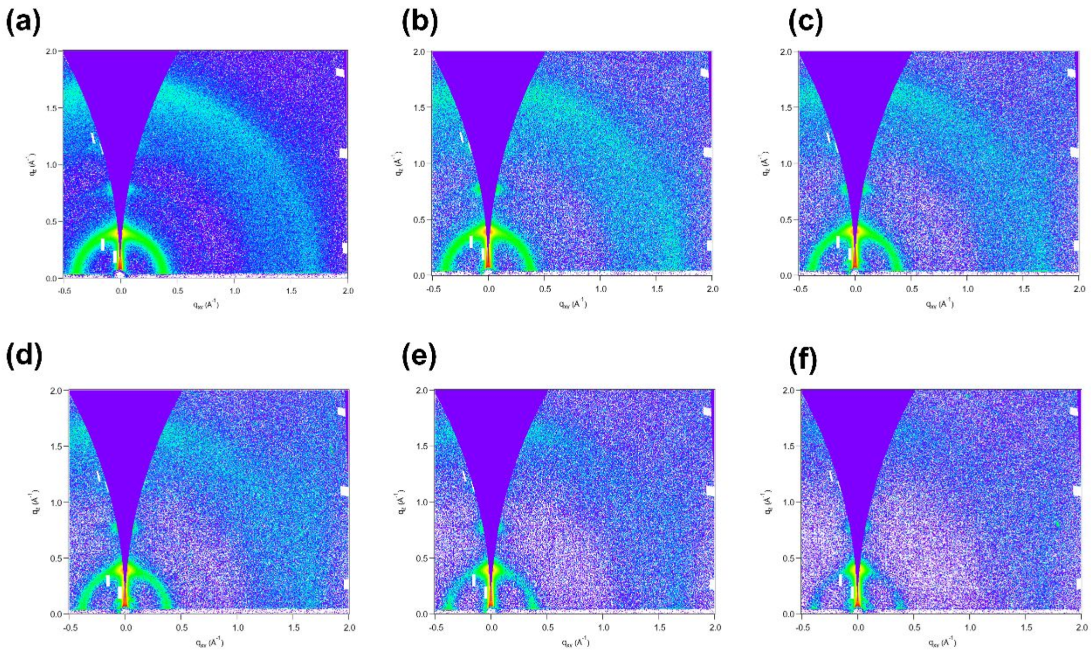

Figure S7. Representative GIWAXS 2D images for P3HT at various film thickness **a)** 80 nm **b)** 51 nm **c)** 38 nm **d)** 30 nm **e)** 20 nm **f)** 13 nm

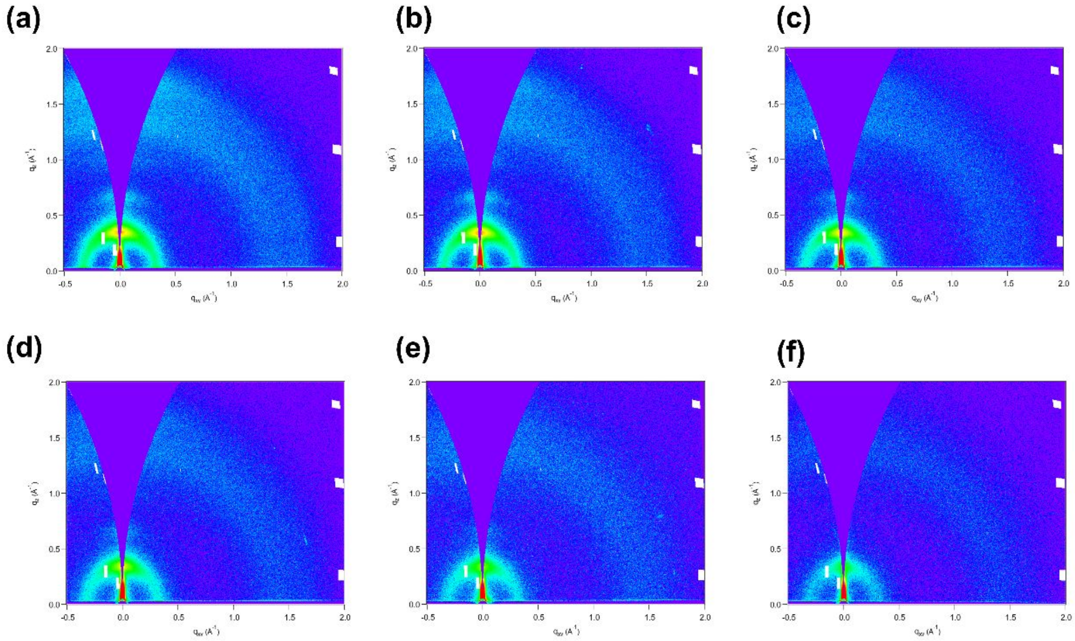

Figure S8. Representative GIWAXS 2D images for PDPPT at various film thickness **a)** 121 nm **b)** 88 nm **c)** 68 nm **d)** 40 nm **e)** 33 nm **f)** 23 nm

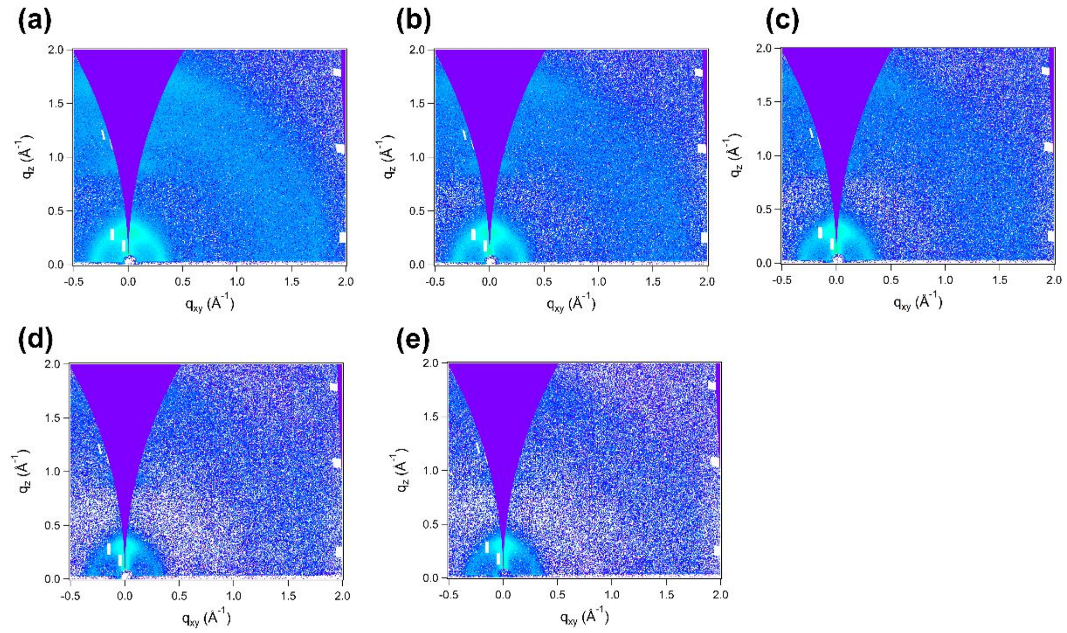

Figure S9. Representative GIWAXS 2D images for PM6 at various film thickness **a)** 126 nm **b)** 69 nm **c)** 50 nm **d)** 23 nm **e)** 13 nm

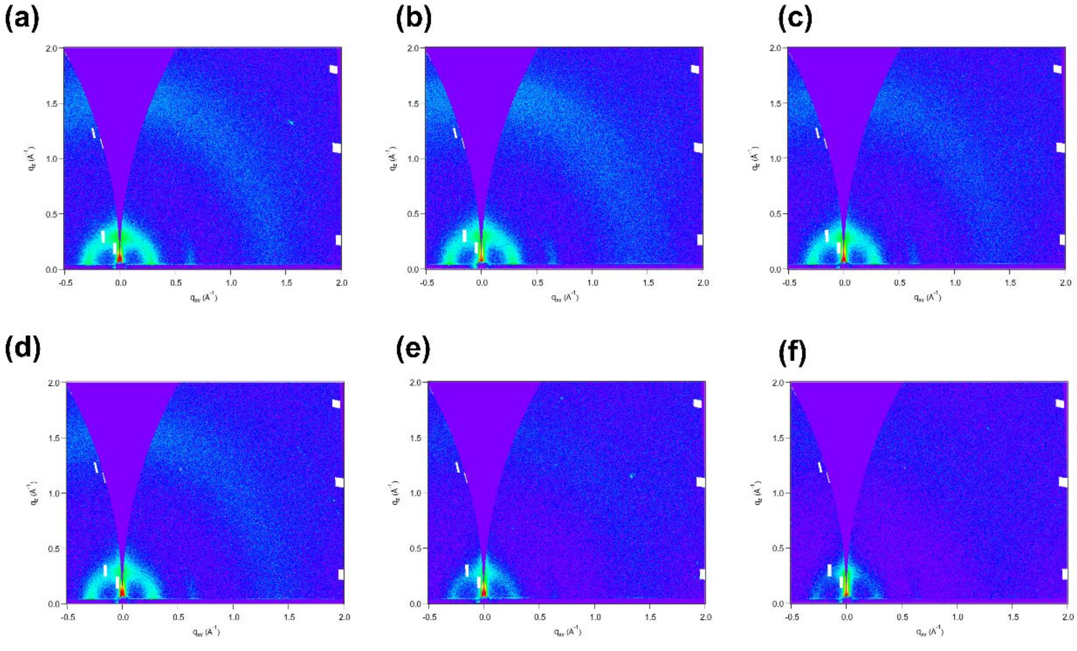

Figure S10. Representative GIWAXS 2D images for PNDI at various film thickness **a)** 63 nm **b)** 55 nm **c)** 44 nm **d)** 37 nm **e)** 16 nm **f)** 12 nm

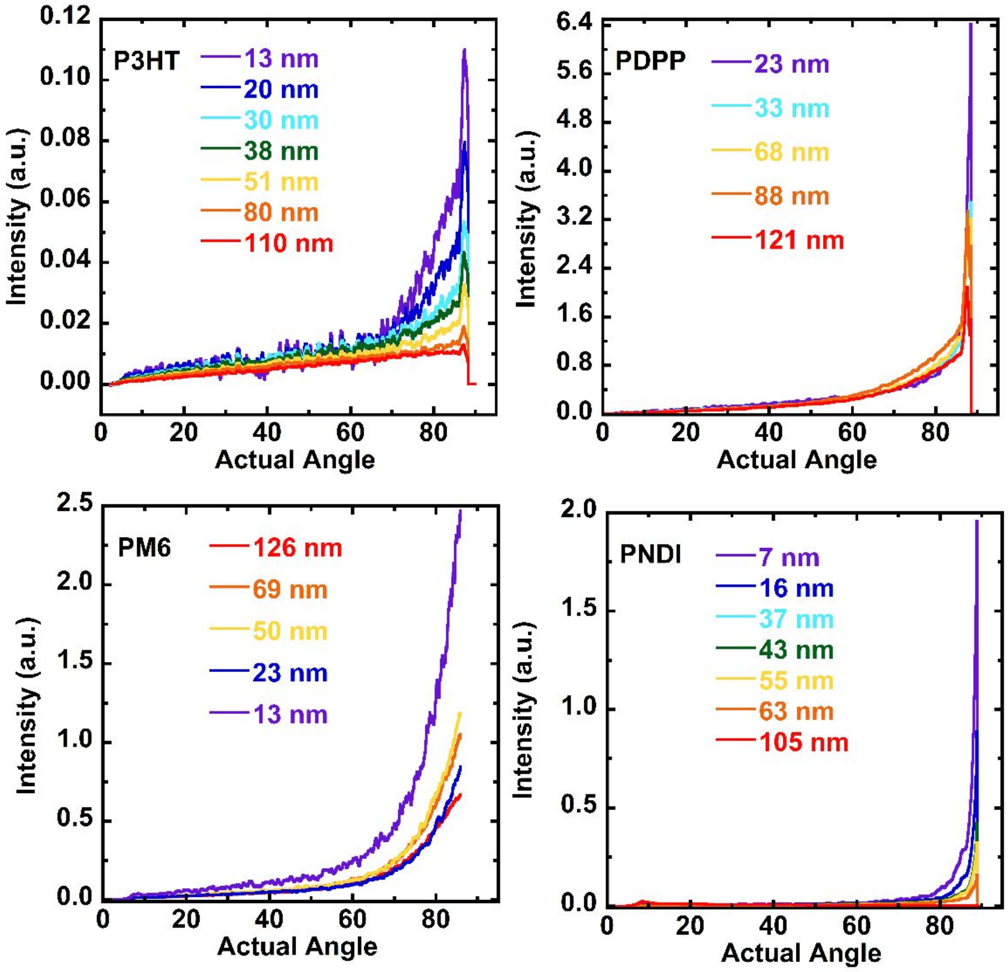

Figure S11. Pole figures for rDOC calculations for P3HT, PDPP, PM6 and PNDI

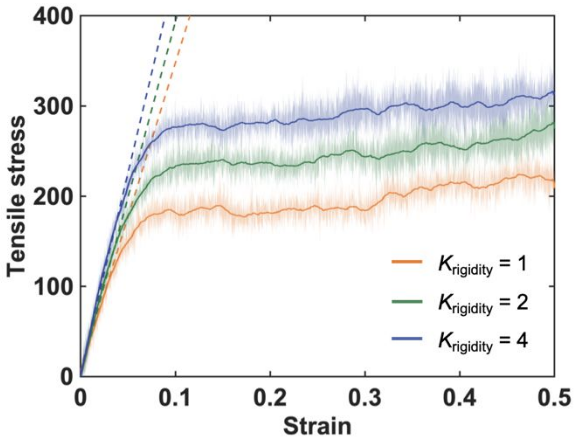

Figure S12. Stress-strain response for bulk polymers with different chain rigidity K_{rigidity} under uniaxial tension. The elastic modulus is determined by linearly fitting the stress-strain data within 4% strain as marked by the dashed lines.

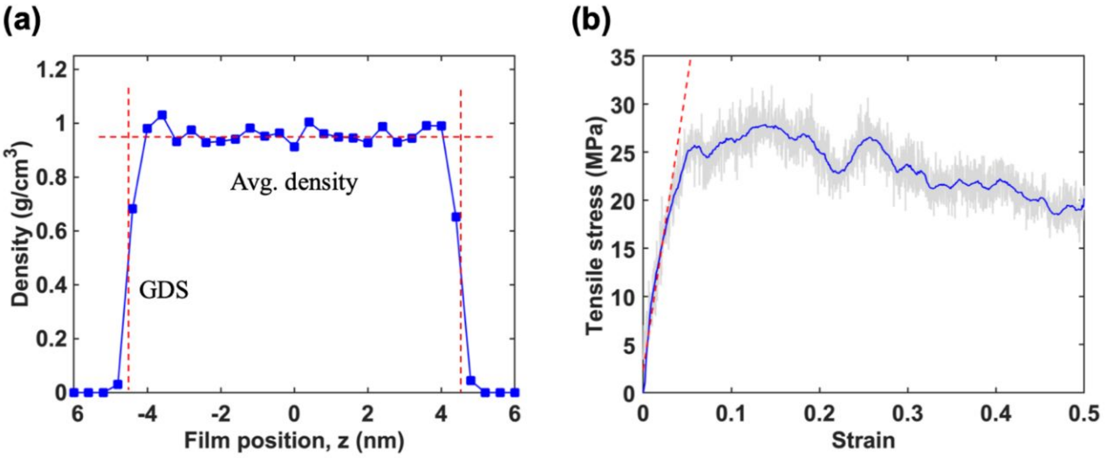

Figure S13. a). Representative density profile along the height of thin film z with a thickness of ~ 10 nm for K_{rigidity} = 1. **b).** Representative stress-strain response for thin film system with a thickness of ~ 10 nm for K_{rigidity} = 1. Dashed line refers to the linear fit of elastic deformation stage for determining the elastic modulus.

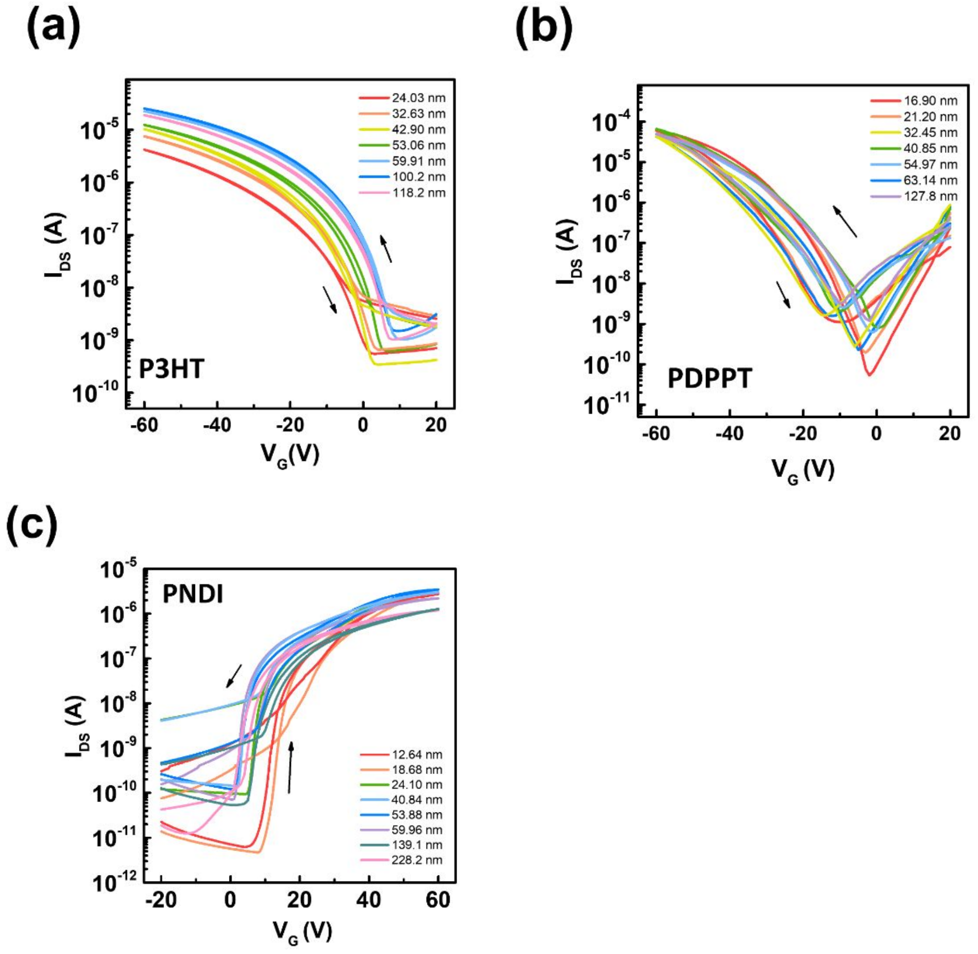

Figure S14. The hysteresis curve of different conditions for **a)** P3HT **b)** PDPPT **c)** PNDI at V_{DS} = -60 V

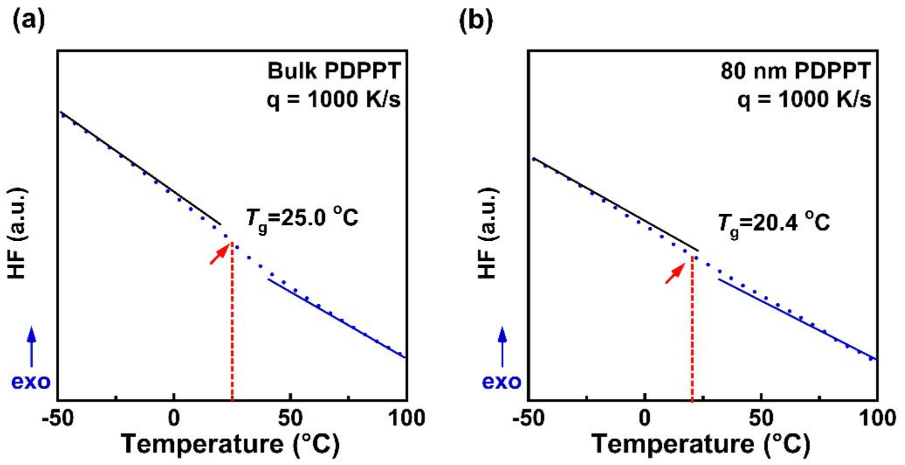

Figure S15. T_g measurements for bulk and 80 nm PDPPT samples at cooling rate of 1000 K/s

Table S1. T_g Values for CG Polymer Models with Different Chain Rigidity ($K_{rigidity}$).

	$K_{rigidity} = 1$	$K_{rigidity} = 2$	$K_{rigidity} = 4$
$T_g^{film \sim 10\ nm}$	434.5 K	472.4 K	530.5 K
$T_g^{film \sim 30\ nm}$	437.3 K	473.4 K	533.3 K
T_g^{bulk}	447.1 K	485.8 K	548.4 K
ΔT_g	~ 12.6 K	~ 13.4 K	~ 17.9 K
